# A Self‐Assembling Flavin for Visible Photooxidation

**DOI:** 10.1002/chem.202201725

**Published:** 2022-07-12

**Authors:** Michele Cariello, Bart Dietrich, Lisa Thomson, Valentina Gauci, Alistair Boyer, Stephen Sproules, Graeme Cooke, Annela Seddon, Dave J. Adams

**Affiliations:** ^1^ School of Chemistry University of Glasgow Glasgow G12 8QQ UK; ^2^ School of Physics, HH Wills Physics Laboratory University of Bristol Tyndall Avenue Bristol BS8 1TL UK

**Keywords:** catalysis, flavin, gel, oxidation, supramolecular

## Abstract

A new flavin‐based gelator is reported which forms micellar structures at high pH and gels at low pH. This flavin can be used for the photooxidation of thiols under visible light, with the catalytic efficiency being linked to the self‐assembled structures present.

## Graphical abstract

**The different micellar and gel states** formed by a flavin‐based gelator can be used as effective and selective photocatalysts under visible light. The self‐assembled structure formed directly translates into a difference in the activity of these structures in the photooxidation reactions.

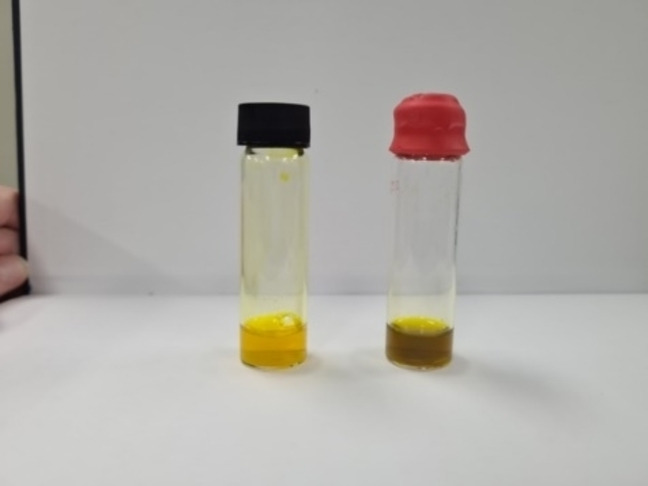

## Introduction

Flavins are widespread redox‐active molecules occurring in nature as co‐factors able to transfer electrons in the respiratory chain.^[1]^ This feature, together with their distinctive optical properties, has made them a useful and highly studied class of materials. Derivatives of riboflavin have been used in a number of applications, such as in fluorescent probes,^[2]^ photocatalysts^[3]^ and batteries.^[4]^ In addition, non‐natural synthetic flavins offer predictable and fine‐tunable redox and optical properties^[5]^ which can provide functional materials with a range of applications.^[6]^

Aggregation of chromophores can be used as a means of tuning the optical and electronic properties of chromophore‐containing systems. One method of inducing aggregation which has the potential for controlling π–π stacking is to form gels from suitably functionalized chromophores.^[7]^ For example, a range of interesting chromophores can be coupled to amino acids and dipeptides to form so‐called low molecular weight gelators. These are molecules which self‐assemble into one‐dimensional structures that entangle to form the matrix of a gel. The self‐assembly often leads to aggregation that can be tuned by the conditions under which the self‐assembly is carried out, resulting in specific changes in optoelectronic and photocatalytic behavior.^[7a]^ For example, the self‐assembly of a single amino acid‐functionalized perylene bisimide results in different structures depending on the pH of the system and the efficiency of this perylene bisimide as part of a photocatalytic system depends on the structures formed.^[8]^ Similar observations have been made for other systems.^[9]^

To the best of our knowledge, only two single flavin‐based low molecular weight gelators have been reported.^[10]^ One was used for the aerobic reduction of olefins.^[10b]^ A flavin block copolymer that forms gels has also been reported.^[11]^ Here, we describe a dipeptide‐functionalized flavin that was demonstrated to form hydrogels using different gelation‐triggers and to be a suitable catalyst for the visible‐light promoted oxidation of thiols.

## Results and Discussion

**Scheme 1:**
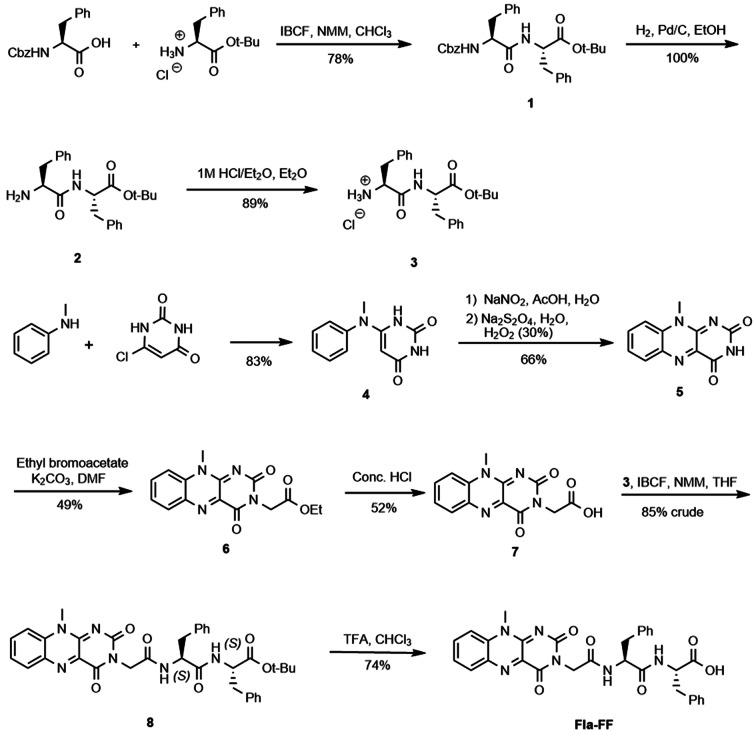
Synthetic route to the gelator **Fla−FF**.

**Fla−FF** (Scheme 1) was prepared by the coupling of compound **7** (prepared by literature procedures^[12]^) with a suitably protected phenylalanine‐phenylalanine dipeptide **3** followed by deprotection.

The flavin core with an appropriate carboxylic acid linker **7** was prepared following an established route in five steps.^[12]^ The dipeptide **1** was prepared by coupling N‐Cbz‐protected (*S*)‐phenylalanine with (*S*)‐phenylalanine *tert*‐butyl ester via a mixed anhydride. Reductive deprotection of the N‐terminus (Pd/C, H_2_) gave the amine **2** that was transformed into its hydrochloride salt **3** using HCl in Et_2_O for ease of handling. The two key components **3** and **7** were joined in another *iso*‐butyl chloroformate‐mediated coupling to yield *tert*‐butyl ester **8**. Finally, acid‐mediated deprotection and recrystallisation yielded the dipeptide functionalized flavin **Fla−FF**.

**Figure 1:**
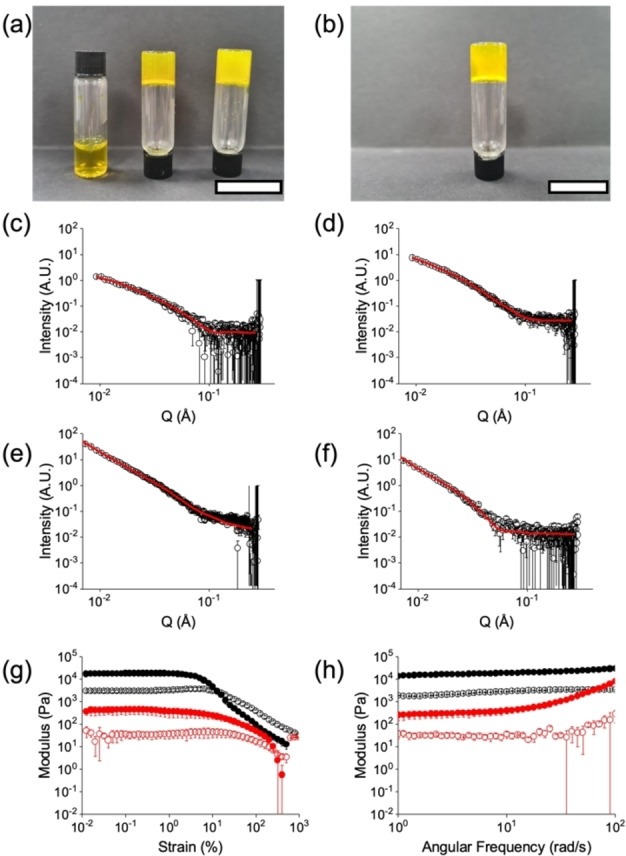
(a) Photographs of (from left to right) solution of **Fla−FF** at high pH, gel of **Fla−FF** at low pH formed using GdL and gel of **Fla−FF** formed by dissolution in DMF followed by addition of water. (b) Photograph of a gel of **Fla−FF** at low pH formed using HCl. For (a) and (b) the scale bar represents 2 cm. Also shown are SAXS data (open circles) and fits (red lines) for (c) a solution of **Fla−FF** at pH 9, fitting to a flexible cylinder; (d) the pH‐triggered gel at pH 3.9 formed using GdL, fitting to a flexible elliptical cylinder; (e) the pH‐triggered gel at pH 4 formed using HCl, fitting to a flexible cylinder combined with a power law; (f) the solvent‐triggered gel, fitting to a flexible elliptical cylinder; (g) rheological strain sweep and (h) frequency sweep for (black) the pH‐triggered gel and (red) the solvent‐triggered gel. Full symbols represent G′ and open circles represent G“.

A solution of **Fla−FF** was prepared at a concentration of 5 mg/mL (Figure 1a, left) by deprotonation of the terminal carboxylic acid with an equimolar amount of sodium carbonate (note that using an excess of a strong base such as sodium hydroxide resulted in decomposition). The resulting solution (at pH 9) contained micellar aggregates; small angle X‐ray scattering (SAXS) data can be best fit to a flexible cylinder model (Figure 1b) with a radius of 3.1 nm, a Kuhn Length of 5.8 nm and a length outside the length scale accessible by this technique, consistent with the presence of wormlike micelles.

A self‐supporting gel was formed on addition of glucono‐δ‐lactone (GdL), which slowly hydrolyses to gluconic acid and results in a decrease in pH (Figure 1a, middle). This slow hydrolysis leads to homogeneous gels being formed.^[13]^ Unfortunately, drying of such systems as required for TEM or SEM often leads to artefactual data. SAXS provides data on the bulk sample without drying and hence is more indicative of the structures present. SAXS shows that the gel state is underpinned by a network of flexible elliptical cylinders, typical for such pH‐triggered gels (Figure 1d),^[14]^ with a radius of 2.5 nm, an axis ratio of 3, a Kuhn length of 15 nm and a length outside the length scale accessible with SAXS. This suggests that the micelles at high pH decrease in radius on protonation, followed by a lateral aggregation giving an apparent increase in ellipticity.

The precursor solution can also be acidified using HCl, which also results in the formation of a gel (Figure 1b). To compare with the GdL‐triggered gel, we carried out SAXS (Figure 1e). The fit to the data is different to the GdL‐triggered gel, best fitting to a flexible cylinder model combined with a power law to take into account the excess scattering at low Q (this is due to the network being formed and is not uncommon for such gels). The radius is significantly larger than the structures formed in the gel phase. The implication of this is that there are different self‐assembled structures formed depending on whether a slow pH change using GdL or whether a fast pH change with HCl is carried out. It is unsurprising that different methods of self‐assembly lead to different aggregation considering that such gels are highly kinetically trapped.^[15]^

Gels were also formed using a solvent‐triggered approach, whereby **Fla−FF** was dissolved in DMF at a concentration of 25 mg/mL and diluted with water to a final concentration of 5 mg/mL. Using this method, a self‐supporting gel was again formed (Figure 1a, right). There is a marked difference in the SAXS data between the gels formed by a pH trigger and a solvent trigger. In the case of the solvent triggered gel, there was an increase in radius from the solution, from 3.0 nm to 5.7 nm, and a very large major axis value of 51.3 nm (Figure 1f). A difference in the underlying structures in the different gel phases is consistent with our previous work;^[16]^ the outcome of the self‐assembly for a specific molecule is strongly affected by the process by which gelation is induced.^[15a]^

In both cases, the rheological properties for the gels are typical for low molecular weight gels, breaking at relatively low strain and having a frequency independent storage (G′) and loss (G“) modulus (Figure 1h). Consistent with our SAXS data, the rheology of the gels is different depending on which gelation trigger is used. Gels triggered using a pH trigger are an order of magnitude stiffer compared to those triggered by solvent (Figure 1g and 1h). The gels also show different breakdown thresholds under strain (Figure 1g).

**Figure 2:**
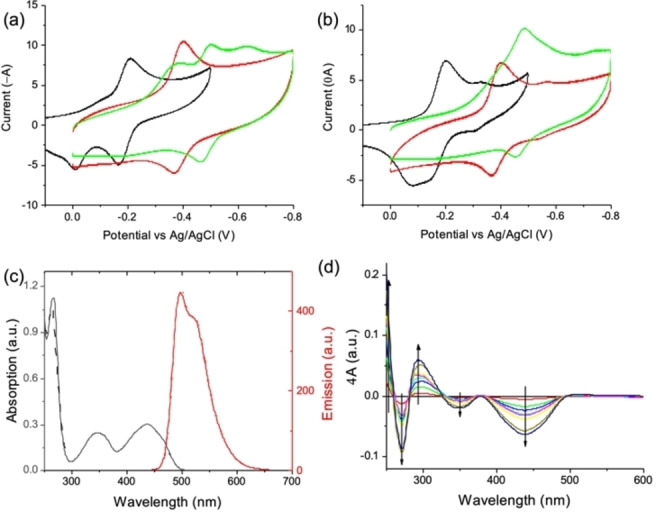
(a) CV traces of flavin **5** in buffered electrolytes; (b) CV traces of **Fla−FF** in buffered electrolytes. For (a) and (b), the green data are for pH 9, the red data for pH 6 and the black data for pH 3; (c) Absorption and fluorescence of **Fla−FF** (solid line) and **5** (dashed lines) of compounds **5** and **Fla−FF** in water at pH 7. The emission spectra were recorded by exciting the samples at 445 nm; (d) SPEC traces of **Fla−FF** in water at pH 7, displayed as difference plots with the trace of the absorption at no applied potential. A reduction potential of ΔV=0.1 V is progressively applied.

The cyclic voltammetry (CV) of compounds **5** (Scheme 1) and **Fla−FF** were recorded at a concentration of 0.1 mM, using buffered electrolyte at pH 3, pH 7 and pH 9 at a concentration of 0.1 M. All solutions were purged with N_2_ prior to use. The CV traces of **5** at different pH are shown in Figure 2a.

At pH 7, a reversible reduction is observed at −0.39 V (vs. Ag/AgCl). This is in good agreement with previously reported results^[17]^ and likely corresponds to a 2e^−^ process (Fl_ox_↔H_2_Fl_red_,).^[18]^ The 2e^−^ process happening at pH 7 is reversible where a clear linearity between the peak currents and the potential sweep rate can be observed (Figure S18, Supporting Information). In line with previously reported data,^[19]^ changing the pH of the solutions significantly alters the electrochemical behavior of flavin **5**, with redox potentials shifting towards more negative values when the acidity decreases and with the loss of reversibility, due to competing protonation/deprotonation reactions.

A similar behavior concerning the peak potential shift as a function of the pH can be seen for **Fla−FF** (Figure 2b). At pH 7, a reduction is observed at −0.39 V (vs. Ag/AgCl), likely corresponding to the same 2e^−^ reduction. The electrochemical reaction is also reversible, as shown in Figure S19, Supporting Information. The presence of a weaker redox event can be seen at more negative potentials. This is likely due to the peptide residue, as it is not observed for parent compound **5**. The electrochemical behavior of **Fla−FF** at pH 3 is like that of flavin **5**, with the oxidation wave splitting into two waves (Figure S19). Also in this case, a weaker redox event is observed at lower potentials. A significant difference from flavin **5** can be noted for the CV trace of **Fla−FF** at high pH, with the presence of one main redox event, although characterised by a high capacitive current, in contrast with the three reduction waves seen for compound **5**. This is possibly due to the alkylation of the imide group (at position N3), which does not allow competing redox reactions promoted by deprotonation (and tautomerization).^[18]^

The absorption and fluorescence spectra of compound **Fla−FF** were recorded in water at a concentration of 0.1 mM and shown in Figure 2c. Both spectra are almost identical to those of parent flavin **5**, indicating that the effect of the peptide chain does not affect the optical properties of the flavin. Three main bands of absorption are observed, peaking at 265 nm, 346 nm, and 434 nm, with a cut‐off of absorption at 500 nm. Solutions of **Fla−FF** at pH 7, 3 and 9 were characterized by spectroelectrochemistry (SPEC) within the same potential window used for the CV analysis. The spectra recorded with no applied potential were almost identical, regardless of the pH, indicating that the oxidized species of **Fla−FF** have similar absorption properties, regardless of their protonation state.

The SPEC traces of **Fla−FF** buffered at pH 7, depicted in Figure 2d as the difference of absorption from a null potential, show the evolution of the absorption spectrum while a reduction potential is applied. The presence of isosbestic points confirms that only two species are involved. These are likely Fl_ox_ and H_2_Fl_red_, with the latter forming while the former gets depleted. The evolution of the reduction process could be observed from the progressive attenuation of the band at 434 nm and the blue shift of the bands at 346 nm and 285 nm. This process is reversible; the original spectrum obtained by applying a positive potential. A similar trend is observed when the pH is adjusted to 9 (Figure S20), while at pH 3 (Figure S19), the SPEC plot is more complex, with the absence of isosbestic points that indicates the presence of more than two species in solution, in good agreement with the CV trace.^[20]^

Irradiation with a 450 nm LED leads to radical formation as shown by electron paramagnetic resonance (EPR) spectrum. The spectral profile and resonance position at g=2.0034 matches previously published flavin radicals.^[21]^ There is no difference in the rate of radical growth on irradiation and little suggestion that there is a significant difference in radical mobility in solution or gel phases (Figure S20).

**Figure 3:**
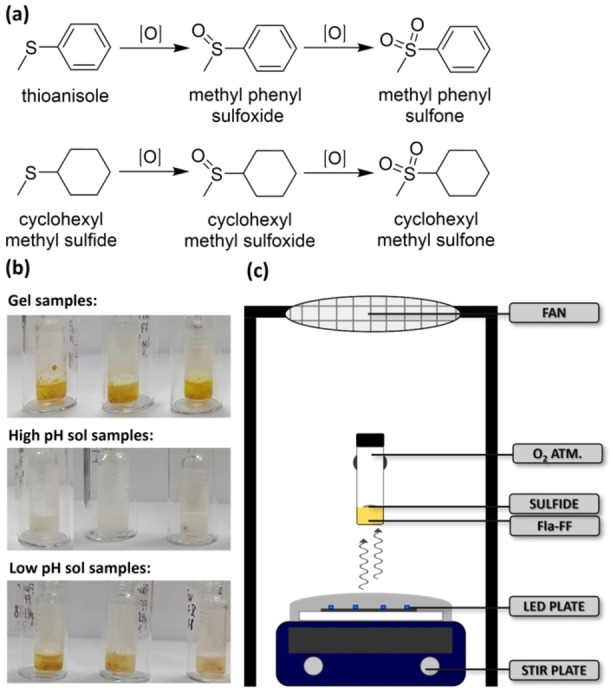
(a) Reaction of sulfides to sulfoxides and sulfones; (b) Photograph of the samples after irradiation with a 445 nm LED for 24 h. (c) Schematic of the reaction setup.

Flavins can catalyze a range of reactions.[^3c^, ^22^] Previous work on the use of a gelling flavin derivative for aerobic hydrogenation of olefins showed that the gel phase provided enhanced catalysis compared to the equivalent non‐gelled catalyst due to the formation of reaction cavities.^[10b]^ Therefore, an interesting question was whether **Fla−FF** could be used as a photocatalyst and whether there would be a difference between activity when used in the solution or gel phase.^[23]^ Here, as proof‐of‐concept, we investigated the photooxidation of a sulfide to a sulfoxide as the main product and a sulfone as the by‐product (Figure 3a). Flavins have been shown to be successful with such oxidations^[24]^ and we highlight that the reaction is complex.^[25]^

The photo‐catalyzed oxidations of thioanisole and cyclohexyl methyl sulfide using **Fla−FF** were investigated under irradiation with blue (445 nm) LEDs. The results for the conversion of the sulfides into the sulfoxides and sulfones are shown in Table 1, with the setup shown in Figure 3c. We compared the **Fla−FF** at high pH (pH 9, in the micellar state) and at low pH (pH 4), both as the gel phase (formed using GdL or HCl as described above, described as gel A or gel B respectively) and in suspension (referred to as a low pH sol, prepared by adding GdL or HCl to the pH 9 sol with stirring, described as sol A or sol B respectively). For both sulfides, conversion to the sulfoxide is highest for **Fla−FF** sol at pH 9. Again, at pH 9 a micellar phase is formed, as not fully dissolved **Fla−FF**. For both the GdL and HCl systems, a slightly reduced conversion is seen for the low pH sol phase compared to the high pH sol phase, with conversion being significantly lower in the gel phase. The sulfone by‐product is the product of over‐oxidation, and its conversion is limited to 5 %. As seen in Table 1, the conversion values follow the same trend as seen for the conversion to the sulfoxide. Interestingly, after completion of the reaction, samples of **Fla−FF** at pH 9 were bleached white whereas the low pH sol and the gel state both at pH 4 remained predominantly yellow (Figure 3b).

**Table 1:** Photooxidation of sulfides using **Fla−FF** in the sol and gel states. [**Fla−FF**]=5 mg/mL; time=24 h, temperature=room temperature. Error bars are calculated from triplicate runs. a reaction carried under argon to exclude O_2_;

	Substrate	Sulfoxide	Sulfone
**Fla−FF** (pH 9)	PhSMe	95 %±1%	5 %±1%
**Fla−FF** (pH 4, sol A)	PhSMe	78 %±14 %	2 %±1%
**Fla−FF** (pH 4, sol B)	PhSMe	86.1 %±4 %	2 %±1%
**Fla−FF** (pH 4, gel A)	PhSMe	37 %±6 %	0 %
**Fla−FF** (pH 4, gel B)	PhSMe	29 %±5 %	0 %
**Fla−FF** (pH 9)	cyHexSMe	81 %±4%	3 % ±0%
**Fla−FF** (pH 4, sol A)	cyHexSMe	53.%±6%	0 %
**Fla−FF** (pH 4, sol B)	cyHexSMe	73 %±8%	1 % ±1%
**Fla−FF** (pH 4, gel A)	cyHexSMe	33 %±3%	0 %
**Fla−FF** (pH 4, gel B)	cyHexSMe	37 %±20 %	0 %
Flavin **7** (pH 9)	PhSMe	45 %±20 %	1 % ±0.5 %
Flavin **7** (pH 9)	cyHexSMe	28 % ±1%	0.5 % ±0%

To understand these systems, several control experiments were undertaken. Very low conversion (<5 %) was observed in the absence of **Fla−FF**, oxygen, or light source. Addition of sodium azide (a singlet oxygen quencher^[26]^) resulted in a significantly lower conversion (15 %) and using 1,3‐diphenylisobenzofuran gave a 40 % conversion to the Diels Alder adduct, both of which suggest a mechanism involving singlet oxygen.^[27]^ No enantioselectivity is observed here despite the chirality of the **Fla−FF**.

The difference between the data for the gel and the low pH sol is quite distinct, despite both systems having the same pH. As described above, there are differences in the underlying self‐assembled structures in the GdL and HCl triggered systems which do translate to slight changes in activity. However, in both cases, the lower activity in the gel phase is presumably due to restricted diffusion into the gel and mixing (diffusion in gels can be restricted due to the gel network). However, for the molecular size of the sulfides used here we would expect diffusion within the network to be unaffected unless there are specific interactions between the network and diffusant,^[28]^ or the diffusant is larger than the pores within the network.^[29]^ However, the bulk gel and sulfide are initially two phases in this case as opposed to a better mixed system when the sols are used and diffusion into or out of gels will be slow). Another explanation is that at high pH the **Fla−FF** is forming a micellar aggregate. Sulfoxidations have been successfully carried out in micellar phases, including with flavinium salts,^[30]^ and can show enhanced reaction rates. We compared the results with those using flavin **7** (Scheme 1) at the same molar concentration at pH 9. Under these conditions, **7** can be dispersed by deprotonation of the carboxylic acid. UV‐Vis spectroscopy shows that the aggregation is similar to **Fla−FF** with a very similar spectrum (Figure S22). However, **7** is much less effective as a photocatalyst, resulting in lower conversions for both thioanisole and cyclohexyl methyl sulfide (Table 1).

## Conclusions

Overall, the difference in self‐assembled structure formed directly translates into a difference in the activity of these structures in the photooxidation reactions. This correlates with recent data elsewhere showing that the activity in a photocatalytic reaction can be varied by changing the self‐assembled structures present even from the same molecule and highlights a different approach to optimizing such systems over generating a library of molecules.^[8]^

In conclusion, we have shown that a flavin‐based gelator can be used to form gels in different ways, with the underlying self‐assembled structures and hence gel properties vary depending on the gelation method. The flavin can be used as a photocatalyst for the oxidation of thiols, with the activity again depending on the self‐assembled structures present.

## Conflict of interest

The authors declare no conflict of interest.

1

## Supporting information

As a service to our authors and readers, this journal provides supporting information supplied by the authors. Such materials are peer reviewed and may be re‐organized for online delivery, but are not copy‐edited or typeset. Technical support issues arising from supporting information (other than missing files) should be addressed to the authors.

Supporting Information

## Data Availability

The data that support the findings of this study are available in the supplementary material of this article.
