# Supplementary material for: A Self‐Assembling Flavin for Visible Photooxidation
Source: Chemistry. 2022 Jul 12;28(49):e202201725. doi: 10.1002/chem.202201725 (PMC9541220; doi:10.1002/chem.202201725)
Supplement: Supplementary file 1 — Supporting Information [file CHEM-28-0-s001.pdf]

Chemistry–A European Journal

Supporting Information

A Self-Assembling Flavin for Visible Photooxidation

Michele Cariello, Bart Dietrich, Lisa Thomson, Valentina Gauci, Alistair Boyer, Stephen Sproules, Graeme Cooke, Annela Seddon, and Dave J. Adams*

Supplementary Information

General Experimental Details

Starting materials and reagents were purchased from either Merck, Fluorochem or TCI and used without further purification. Unless otherwise specified, reactions were carried out in open air. Mass spectrometry was obtained from the mass spectrometry service at the University of Glasgow, using a Bruker MicroTOF-Q. NMRs were recorded on either Bruker Avance III 400 or Bruker Avance III 500 spectrometers. The ^{1}H and ^{13}C spectra were recorded at 400 or 500 MHz and 100 or 125 MHz, respectively, with chemical shift values in ppm relative to residual solvent peaks: 7.26 (^{1}H) and 77.16 (^{13}C) for chloroform, 2.50 (^{1}H) and 39.52 (^{13}C) for DMSO. Coupling constants, *J*, are reported in Hz and the following abbreviations are used to label the multiplicities: s, singlet; d, doublet; t, triplet; m, multiplet and br, broad. NMR spectra were analysed using Mestrenova 14.2 or TopSpin 4.0.9. Melting points were recorded on a SMP-10 Stuart Scientific melting point machine. Melting points are uncorrected. Compound names were generated using Marvin Sketch 21.4 (www.chemaxon.com).

UV-vis absorption spectra were recorded using a Shimadzu UV-3600 spectrophotometer, at a concentration of 1.0×10^{-2} mM. Cyclic voltammetry was performed using a CH Instruments Electrochemical Workstation (CHI 440a), Austin, TX, USA. Samples were analysed in solutions of buffered electrolytes at 1.0 mM concentrations with a scan rate of 0.1 V/s, unless otherwise specified. A glassy carbon working electrode, a Pt wire counter electrode and a Ag/AgCl (3.0 M KCl) reference electrode were used. The buffers were freshly made using deionised water and H_3PO_4/NaH_2PO_4 for pH 3, NaH_2PO_4/Na_2HPO_4 for pH 7 and Na_2CO_3/$NaHCO_3$ for pH 9, respectively, all at 0.1 M concentration. All solutions were purged with N_2 prior to use. The spectro-electrochemistry was performed using a combination of the two instruments above. Absorption spectra of the samples in buffered solutions at 1.0 mM concentrations were recorded one minute after the desired potential was applied. A BASi spectroelectrochemical kit made of a quartz cuvette, a Pt mesh working electrode, a Pt wire counter electrode and an Ag/AgCl (3.0 M KCl) reference electrode were used. The spectra were reported as a difference with those at null potential. Qualitative fluorescence spectroscopy was performed using a Shimadzu RF-5301PC spectro-fluorimeter. All the spectroscopic and electrochemical data were processed using Origin 2020 software suite.

pH-triggered Gels. Fla-FF and an equimolar amount of Na_2CO_3 were dissolved in deionised water to produce a solution of 5 mg/mL with respect to **Fla-FF**. This was stirred overnight to give a homogeneous solution. The pH was adjusted to 9 ± 0.1 using 0.1 M Na_2CO_3 as required. 2 mL of this solution was then pipetted into a Sterilin vial containing GdL (16 mg), stirred briefly with a spatula to dissolve the GdL and then left overnight to allow gelation to occur.

Alternatively, to the stock solution of **Fla-FF**, 1 M HCl was added (18 µL per 1 mL of stock solution) and the sample allowed to stand.

Solvent-triggered Gels. A 25 mg/mL stock solution was prepared by dissolving **Fla-FF** in DMF. Once dissolved, 0.4 mL of stock solution was pipetted into a metal cup. 1.6 mL of deionised water was then quickly pipetted into the metal cup, giving a final **Fla-FF** concentration of 5 mg/mL in a 20:80 solvent:water ratio. The metal cup was then sealed with Parafilm and left overnight to allow gelation to occur.

pH Measurement. A calibrated FC200 Hanna pH probe was used to measure the pH of both solutions and gels. The accuracy stated by the supplier is ± 0.1.

pK_a Titrations. To determine the apparent pK_a of 5 mg/mL **Fla-FF**, a titration with 0.1 M HCl was carried out in a water bath set to 25 °C. 2 mL of **Fla-FF** solution was titrated with 5 µL portions of 0.1 M HCl until a final pH of 3 was reached. To prevent localised gelling around the drop of HCl, the solutions were stirred after the addition of each HCl aliquot until the pH read as stable.

Overnight pH Measurements. Using a custom-built pH logger, pH was measured overnight whilst gelling occurred. 2 mL of 5 mg/mL **Fla-FF** solution was added to GdL as described previously and then a calibrated FC200 Hanna pH probe was quickly placed in the sample and the logger started. The pH was recorded every minute over a 16-hour period.

Rheology. All rheological measurements were carried out using an Anton Paar Physica MCR101 rheometer. Viscosity measurements were performed using a 75 mm cone geometry (CP75) with gap distance between the geometry and the plate set to 0.05 mm and temperature set to 25 °C. Samples were poured onto the rheometer flat plate for measurement and fresh sample used for each run. Viscosity measurements were performed in triplicate, and values averaged with error bars representing the standard deviation between the repeats.

For strain and frequency measurements, measurements were performed in Sterilin vials for pH triggered gels and metal cups for solvent triggered gels. This was due to separation of the solvent triggered gels in Sterilin vials. All strain and frequency measurements were performed using a vane geometry with gap distance between the geometry and the bottom of the sample vial set to 1.8 mm and temperature set to 25 °C. Measurements were performed in triplicate, and values averaged with error bars representing the standard deviation between the repeats. Frequency sweeps were performed under a strain of 0.1 %. Strain sweeps were performed at an angular frequency of 10 rad/s.

For overnight time sweep measurements, 2 mL of 5 mg/mL **Fla-FF** solution was added to 8 mg/mL GdL in a Sterilin vial and briefly stirred with a spatula to dissolve the GdL. This was then quickly poured onto the rheometer flat plate. Measurements were performed at 25 °C using a 50 mm sandblasted parallel plate (PP50/S) and a measuring gap of 0.8 mm for 16 hours to allow for the monitoring of gel formation.

Electron Paramagnetic Resonance (EPR) Spectroscopy. EPR data were recorded on a Bruker ELEXSYS E500 spectrometer equipped with an ER 4102ST-O optical transmission resonator. Samples were transferred into soda glass capillary tubes of 2 mm diameter, sealed at one end, and filled to a height of 20 mm. The sample was irradiated at 450 nm during data acquisition.

Photocatalysis. A solution of 5 mg/mL **Fla-FF** in H_2O was prepared with 1 equivalent 0.1 M Na_2CO_3 and left to stir overnight. The solution was adjusted to pH 9 with 0.1 M Na_2CO_3. This solution was used for the high pH sol samples. Low pH sol (HCl) samples were prepared by adjusting the pH 9 solution to pH 4 with 2 M HCl while stirring vigorously. Low pH sol (GdL) samples were prepared by adding the pH 9 solution to 8 mg/mL GdL and left to stir overnight.

GdL gel samples were prepared by adding the pH 9 solution to 8 mg/mL GdL and left to set overnight. HCl gel samples were prepared by adding 1 M HCl to pH 9 solution (18 µL per 1 mL of **Fla-FF** solution) and were left to set overnight.

Samples of 250 µL **Fla-FF** (2.15 µmol) were prepared in a glass vial (height = 2.3 cm, Ø = 1 cm). Thioanisole (14 mg, 113 µmol) was added to the **Fla-FF** sample. The vial was placed in a larger glass vial (volume = 19 cm^3, height = 6 cm, Ø = 2 cm), and flushed with O_2 gas (estimated 800 µmol). The sealed vial was placed 8 cm above an LED plate (4 × 4, 20 mm spaced grid of Oslon Square 445 nm LEDs, at 1.5 A), and left to stir at 100 rpm while being cooled with a large fan sufficient to maintain the reaction vials below 30 °C. After 24 hours, H_2O and $CDCl_3$ were added to the reaction mixture and stirred for 10 mins. The organic layer was isolated and transferred to an NMR tube. The conversion of the reaction was calculated by ^1H NMR spectroscopy using the ratio of integrals between the methyl singlet peaks of thioanisole (2.50 ppm), methyl phenyl sulfoxide (2.75 ppm), and methyl phenyl sulfone (3.10 ppm).

The same procedure was used for the photooxidation of cyclohexyl methyl sulfide, adding 15 mg (113 µmol) of the sulfide to the **Fla-FF** sample. The conversion was calculated by using the ratio of integrals between the methyl singlet peak of cyclohexyl methyl sulfide (2.10 ppm), sulfoxide (2.50 ppm), and sulfone (2.80 ppm).

In all cases, triplicate runs were carried out for each **Fla-FF** condition. The values of the three runs were used to calculate the average conversion values and the standard deviation values that are used to define the error bars.

Photocatalyst preparation for control experiments. A solution of 5 mg/mL **Fla-FF** in H_2O with 0.1 M Na_2CO_3 (1 equivalent) was prepared. The solution was left to stir overnight to ensure dissolution. The solution was then pH adjusted to pH 9 with 0.1 M Na_2CO_3.

Samples of 250 µL **Fla-FF** (2.15 µmol) were prepared in a glass vial (height = 2.3 cm, Ø = 1 cm). The substrate (113 µmol) was added to the **Fla-FF** sample. The vial was placed in a larger glass vial (volume = 19 cm^3, height = 6 cm, Ø = 2 cm). For the control experiment with no O_2, the vial was flushed with Argon gas (estimated 800 µmol). For the other control experiments, the vial was flushed with O_2 gas. The vial for control experiment in the dark was covered in foil and left to stir at 100 rpm. The vials for the other control experiments were placed 8 cm above an LED plate and left to stir at 100 rpm while being cooled with a large fan sufficient to maintain the reaction vials below 30 °C. After 24 hours, H_2O and $CDCl_3$ were added to the reaction mixture and stirred for 10 mins. The organic layer was isolated and transferred to an NMR tube. For thioanisole, the conversion of the reaction was calculated by ^1H NMR spectroscopy using the ratio of integrals between the methyl singlet peaks of the sulfide (2.50 ppm), sulfoxide (2.75 ppm), and sulfone (3.10 ppm). For 1,3-diphenylisobenzofuran, the conversion of the reaction was calculated by ^1H NMR spectroscopy using the ratio of integrals between the triplet peaks at 7.30 ppm and 7.37 ppm.

Photocatalyst preparation for sulfoxidation reactions with Fla7. A solution of 2.5 mg/mL **Fla7** in H_2O with 0.1 M Na_2CO_3 (1 equivalent) was prepared. Complete dissolution was achieved in 5 minutes. The solution was pH adjusted to pH 9 with 0.1 M Na_2CO_3.

Samples of 250 µL **Fla7** (2.15 µmol) were prepared in a glass vial (height = 2.3 cm, Ø = 1 cm). The sulfide (113 µmol) was added to the **Fla7** sample. The vial was placed in a larger glass vial (volume = 19 cm^3, height = 6 cm, Ø = 2 cm), and flushed with O_2 gas (estimated 800 µmol). The sealed vial was placed 8 cm above an LED plate and left to stir at 100 rpm while being cooled with a large fan sufficient to maintain the reaction vials below 30 °C. After 24 hours, H_2O and $CDCl_3$ were added to the reaction mixture and stirred for 10 mins. The organic layer was isolated and transferred to an NMR tube. The conversion of the reaction was calculated by ^1H NMR spectroscopy using the ratio of integrals between the methyl singlet peaks of the sulfide, sulfoxide, and sulfone.

Test of enantioselectivity. A solution of 5 mg/mL **Fla-FF** in H_2O with 0.1 M Na_2CO_3 (1 equivalent) was prepared. The solution was left to stir overnight to ensure dissolution. The solution was then pH adjusted to pH 9 with 0.1 M Na_2CO_3.

A 1 mL sample of **Fla-FF** (8.6 µmol) was prepared in a glass vial (volume = 19 cm^3, height = 6 cm, Ø = 2 cm). The sulfide (452 µmol) was added to the **Fla-FF** sample. The vial was flushed with O_2 gas (estimated 800 µmol). The sealed vial was placed 8 cm above an LED plate and left to stir at 100 rpm while being cooled with a large fan sufficient to maintain the reaction vials below 30 °C. After 24 hours, H_2O and $CDCl_3$ were added to the reaction mixture and stirred for 10 mins. The organic layer was isolated and transferred to an NMR tube. The conversion of the reaction was calculated by ^1H NMR spectroscopy using the ratio of integrals between the methyl singlet peaks of the sulfide, sulfoxide, and sulfone.

Crude compound was analysed by chiral HPLC column (Chiralpak OD-H, 0.46 Ø × 25 cm, 10% iPrOH/hexane, 2 cm^3min^{-1}, λ = 254 nm, tR = 5.7 mins and 6.5 mins). This scaled-up reaction gave a conversion of 56% to the sulfoxide and 2% to the sulfone and showed no enantioselectivity.

Synthetic procedures

6-[Methyl(phenyl)amino]-1,2,3,4-tetrahydropyrimidine-2,4-dione **4**[1]

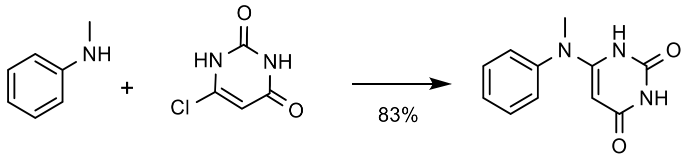

4

N-Methylaniline (3.00 mL, 27.7 mmol) and 6-chlorouracil (2.03 g, 13.8 mmol) were stirred under N_2 for 5 minutes at 180 °C. The mixture was then allowed to cool to room temperature and the resulting precipitate was triturated using a mixture of methanol/diethyl ether (1/5, v/v) to give the title compound **4** as a white solid (2.50 g, 83%). mp = 298 – 299 °C (lit. 302 °C[1]). δ_H (400 MHz, DMSO) 10.43 (1 H, s, NH), 10.27 (1 H, s, NH), 7.48 – 7.42 (2 H, m, H_{Ar}), 7.35 – 7.30 (1 H, m, H_{Ar}), 7.29 – 7.25 (2 H, m, H_{Ar}), 4.26 (1 H, d, *J* 1.4, CO-CH-N), 3.25 (3 H, s, NCH_3). δ_C (100 MHz, DMSO) 163. 7 (C=O), 155.0 (C=O), 151.2 (N-C-NH), 144.1 (N-C_{Ar}), 129.8 (C_{Ar}), 126.9 (C_{Ar}), 126.4 (C_{Ar}), 78.3 (CO-CH). m/z (ESI) 240 ([M+Na]$^+$), 457 ([2M+Na]$^+$).

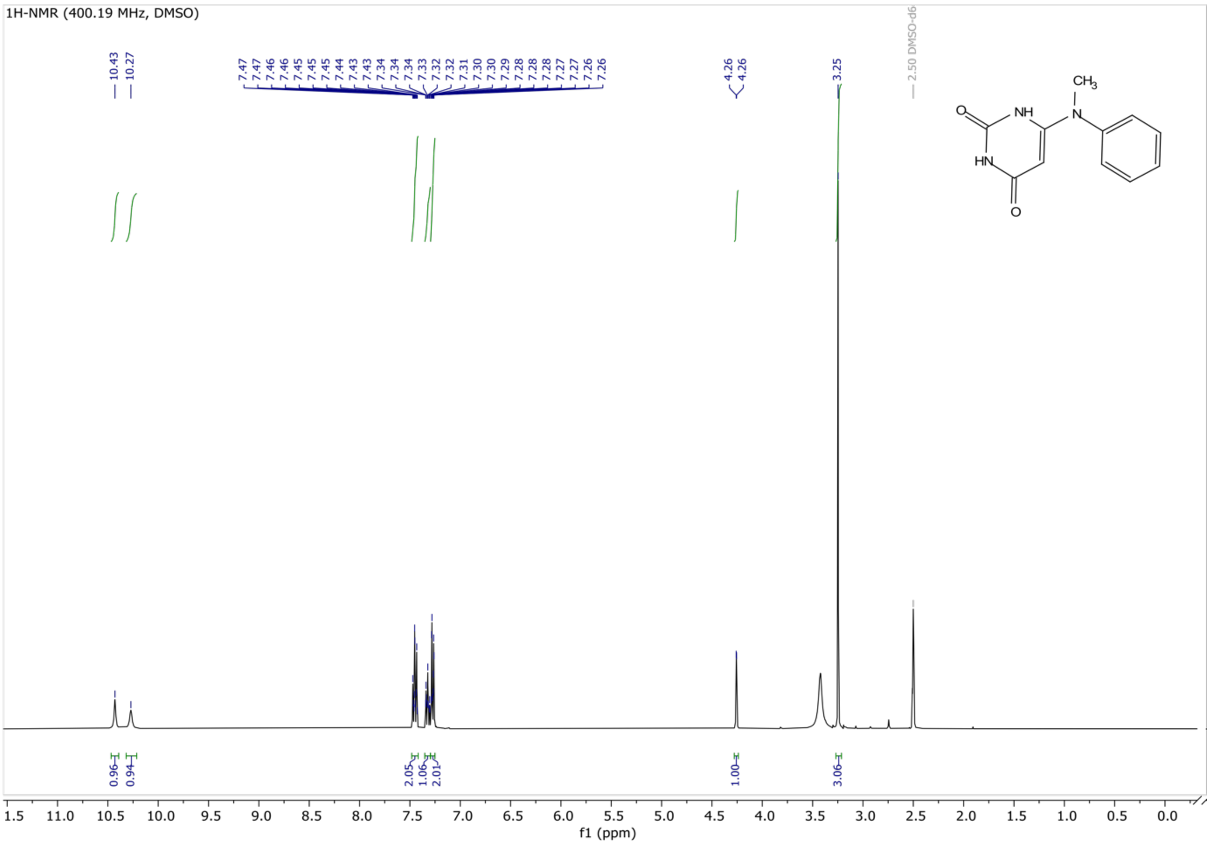

Figure S1. ^{1}H NMR spectrum of compound **4**.

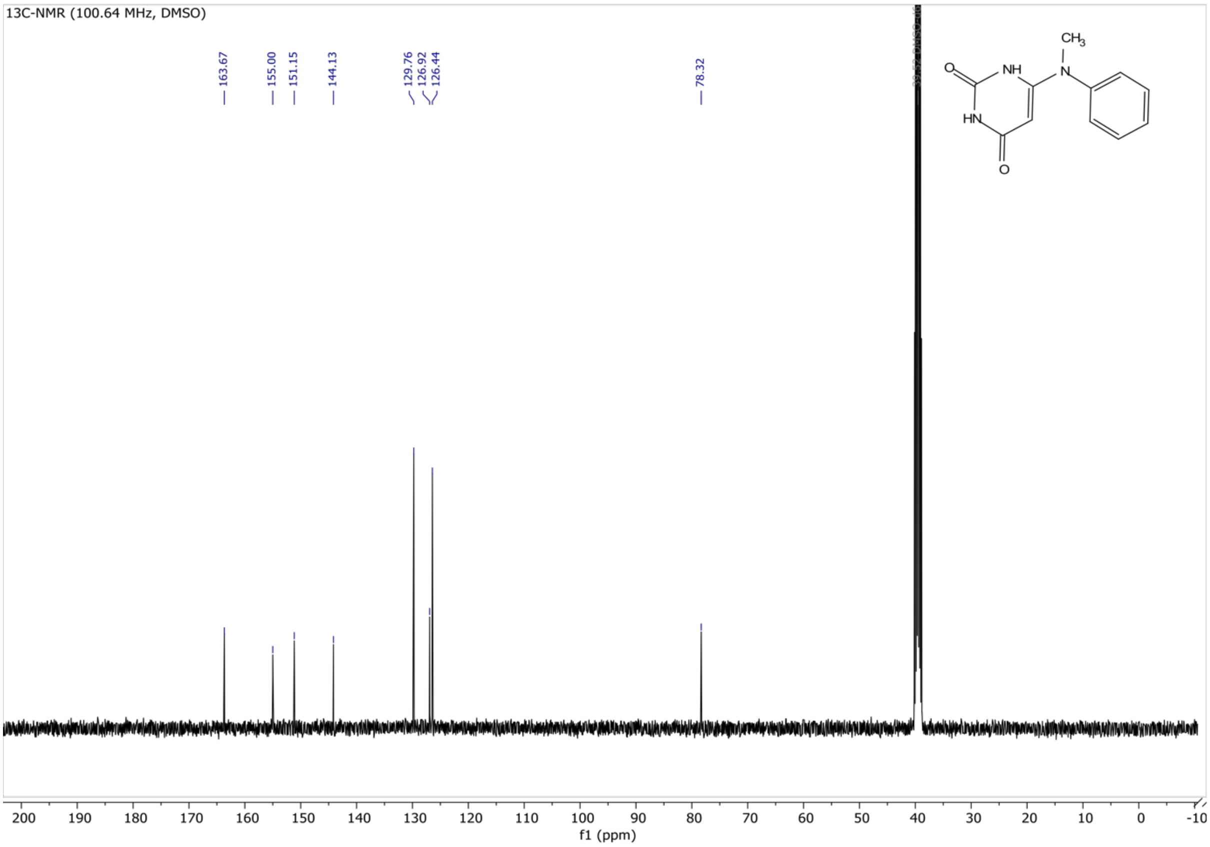

Figure S2. ^{13}C NMR spectrum of compound **4**.

10-Methyl-2H,3H,4H,10H-benzo[g]pteridine-2,4-dione **5**[1]

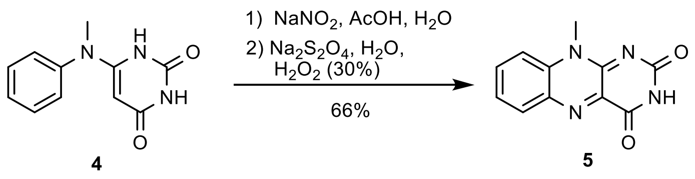

Sodium nitrite (3.97 g, 57.6 mmol) was added to a solution of **4** (2.50 g, 11.5 mmol) in acetic acid (100 mL). The resulting mixture was stirred at room temperature for 3 hours, then it was poured into water (200 mL) and stirred for further 2 hours. The resulting precipitate was collected by vacuum filtration, washed with water (50 mL) and dried under vacuum. The yellow solid was added to a solution of sodium dithionite (6.00 g, 34.5 mmol) in water (100 mL). The mixture was stirred at room temperature for 3 hours, then an aqueous solution of 30% hydrogen peroxide (20 mL) was slowly added. The cloudy mixture was stirred at room temperature for 18 hours. The yellow solid was collected by vacuum filtration and washed with water (50 mL) and cold methanol (20 mL) to afford the title compound **5** as a yellow solid (1.73 g, 66%). mp > 300 °C (lit. 352 °C[1]). δ_H (400 MHz, DMSO) 11.39 (1 H, s, N<u>H</u>), 8.15 – 8.11 (1 H, m, <u>H</u>$_{Ar}$), 7.98 – 7.92 (2 H, m, <u>H</u>$_{Ar}$), 7.69 – 7.63 (1 H, m, <u>H</u>$_{Ar}$), 3.98 (3 H, s, NC<u>H</u>$_3$). m/z (ESI) 251 ([M+Na]$^+$), 479 ([2M+Na]$^+$).

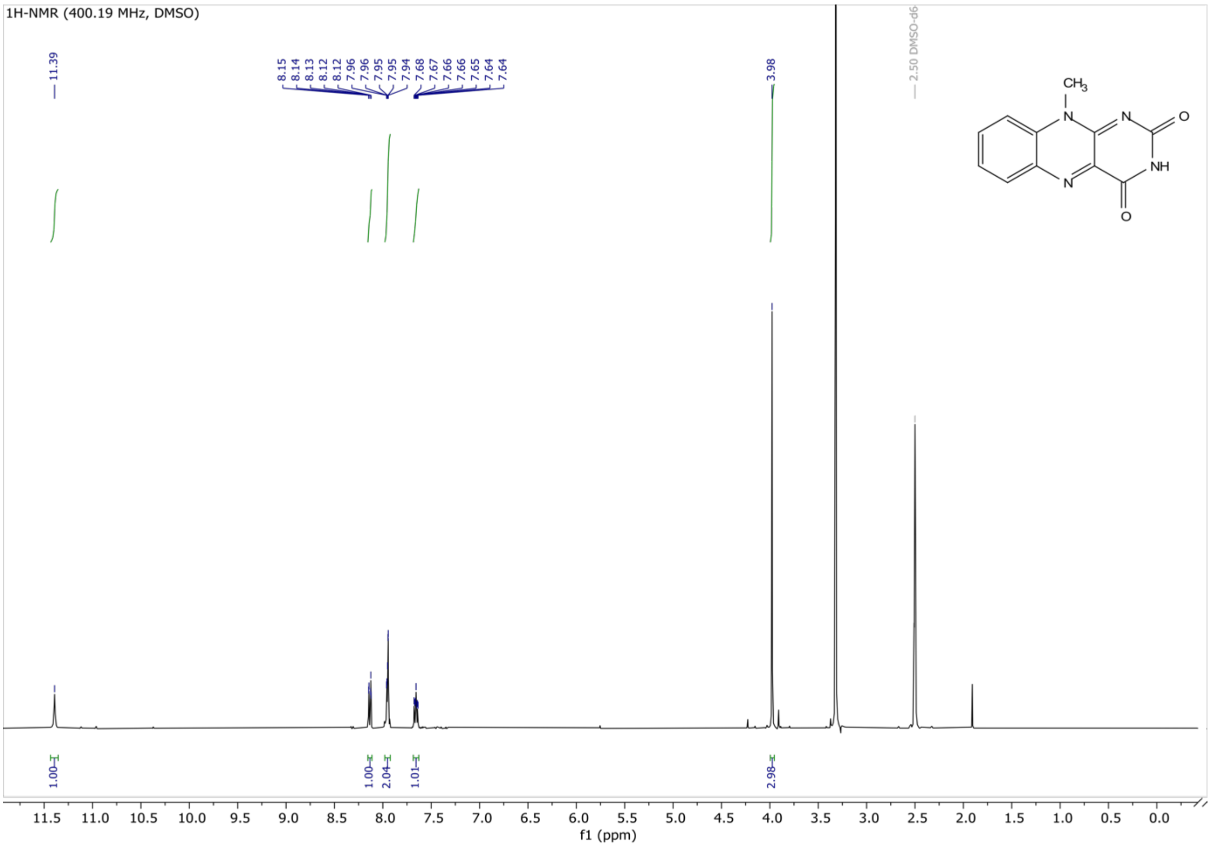

Figure S3. ^{1}H NMR spectrum of compound **5**.

Ethyl 2-{10-methyl-2,4-dioxo-2H,3H,4H,10H-benzo[g]pteridin-3-yl}acetate **6**[2]

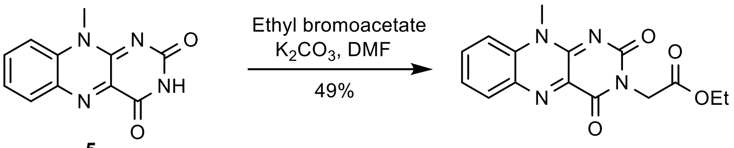

5 6

Potassium carbonate (1.60 g, 4.95 mmol) was added to a solution of **5** (1.00 g, 4.13 mmol) in anhydrous dimethylformamide (DMF, 200 mL), under N_2. The resulting mixture was stirred at 80 °C for 0.5 hours, then ethyl bromoacetate (2.3 mL, 20.7 mmol) was added. After 24 hours, the solvent was evaporated under reduced pressure and the residue was dissolved in dichloromethane (100 mL) and saturated ammonium chloride (100 mL). The organic layer was separated, and the aqueous layer was washed with dichloromethane (2 × 100 mL). The combined organic extracts were washed with water (150 mL) and brine (150 mL), dried over magnesium sulphate, and filtered. The solvent was evaporated under reduced pressure and the residue was triturated using diethyl ether to afford the title compound **6** as a bright yellow solid (0.630 g, 49%). mp = 233 – 235 °C (lit. 235 – 237 °C[1]). δ_H (500 MHz, $CDCl_3$) 8.33 (1 H, dd, *J* 8.5, 1.5, $\underline{H}_{Ar}$), 7.93 (1 H, ddd, *J* 8.5, 7.1, 1.5, $\underline{H}_{Ar}$), 7.69 – 7.63 (2 H, m, $\underline{H}_{Ar}$), 4.86 (2 H, s, $NC\underline{H}_2$), 4.23 (2 H, q, *J* 7.2, $C(O)OC\underline{H}_2$), 4.15 (3 H, s, $NC\underline{H}_3$), 1.29 (3 H, t, *J* 7.2, CH_2-$C\underline{H}_3$). δ_C (125 MHz, $CDCl_3$) 167.8, ($\underline{C}$OOEt), 159.3 (N$\underline{C}$O), 155.0 (N$\underline{C}$O), 149.7 ($\underline{C}_{Ar}$), 137.1 ($\underline{C}_{Ar}$), 136.2 ($\underline{C}_{Ar}$), 135.9 ($\underline{C}_{Ar}$), 133.6 ($\underline{C}_{Ar}$), 133.5 ($\underline{C}_{Ar}$), 126.9 ($\underline{C}_{Ar}$), 115.4 ($\underline{C}_{Ar}$), 61.8 ($N\underline{C}H_2$), 43.1 ($N\underline{C}H_3$), 32.3 ($C(O)O\underline{C}H_2$), 14.3 (CH_2-$\underline{C}H_3$). m/z (ESI) 337 ($[M+Na]^+$), 651 ($[2M+Na]^+$).

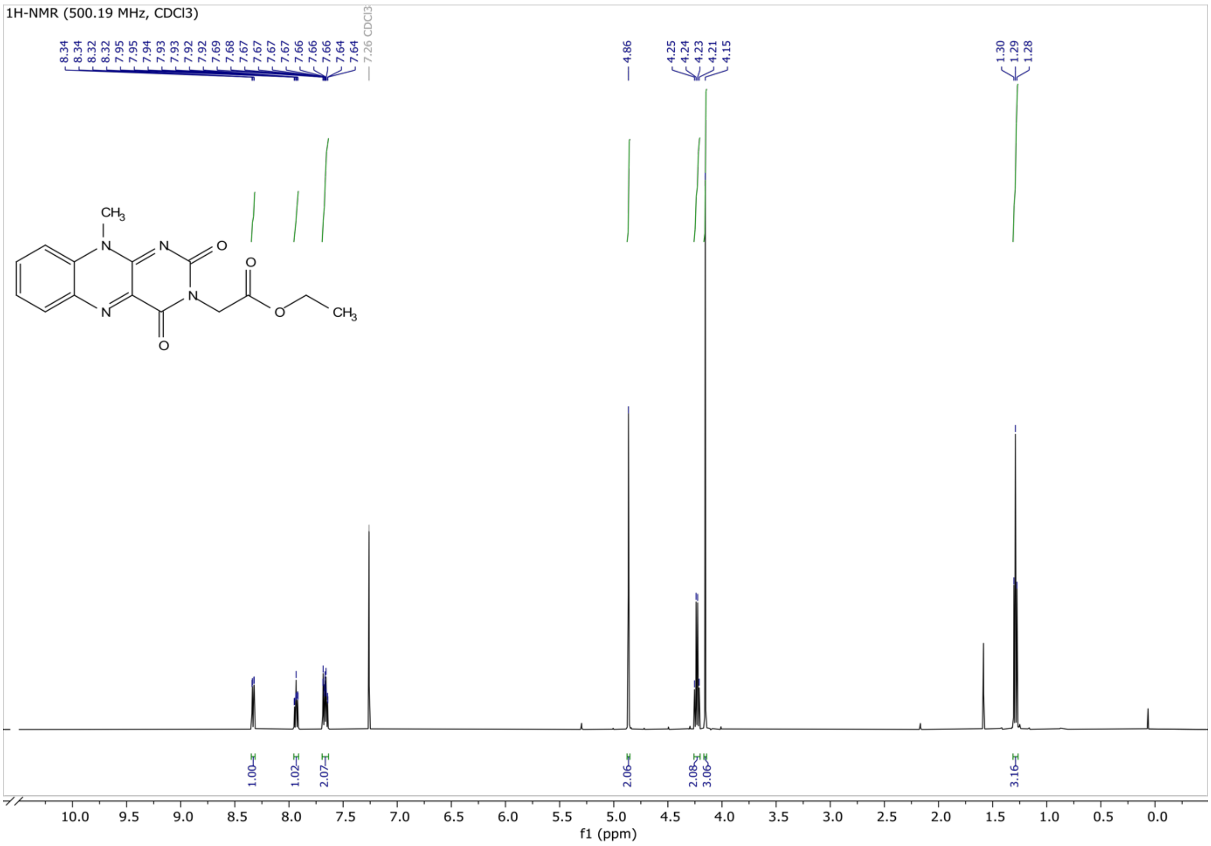

Figure S4. ^{1}H NMR spectrum of compound **6**.

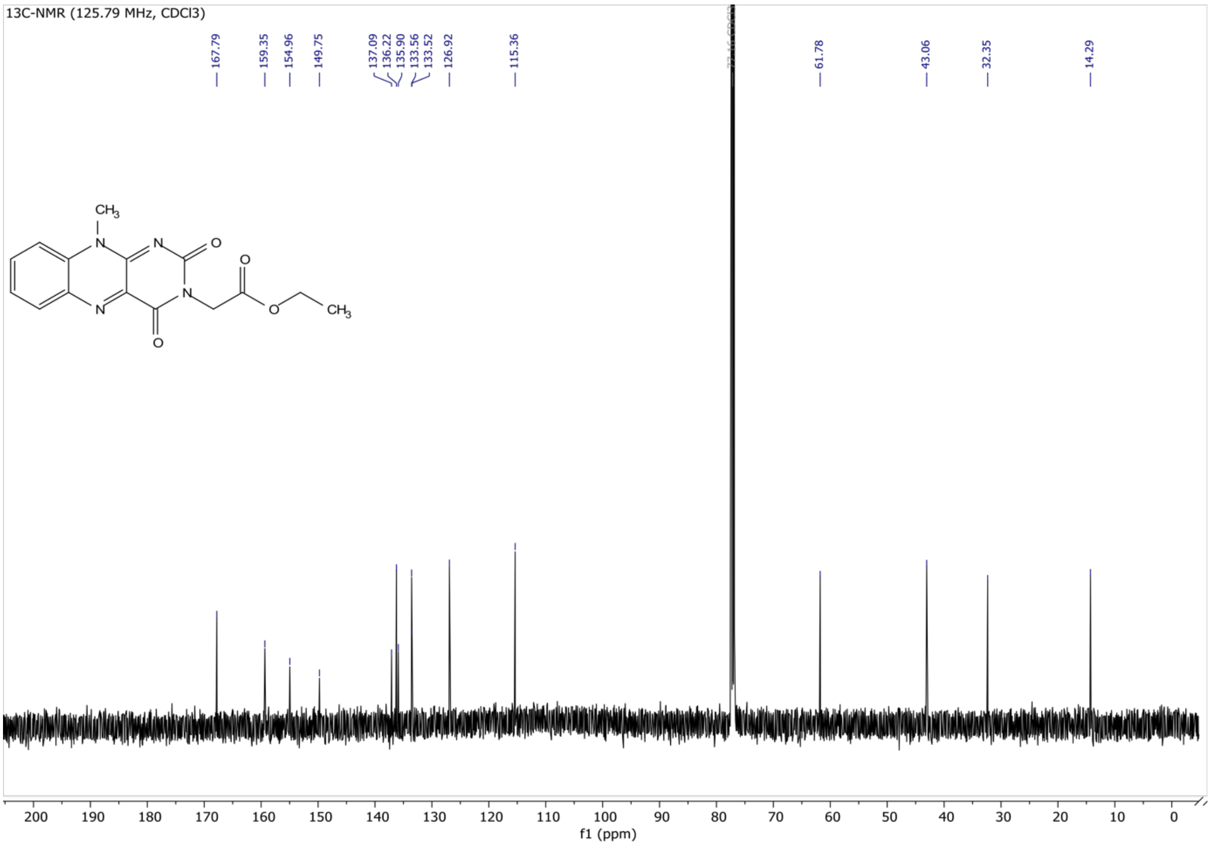

Figure S5. ${}^{13}C$ NMR spectrum of compound **6**.

2-{10-Methyl-2,4-dioxo-2H,3H,4H,10H-benzo[g]pteridin-3-yl}acetic acid **7**[2]

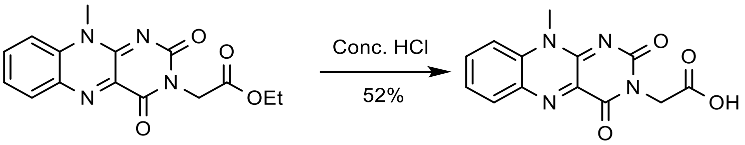

6 **7**

A solution of **6** (0.630 g, 2.00 mmol) in concentrated hydrochloric acid (10 mL) was stirred at 90 °C for 1 hour. The mixture was then allowed to cool to room temperature and poured into ice-water. The resulting suspension was allowed to warm up to room temperature while stirring. The solids were then collected by vacuum filtration and washed with water (50 mL), methanol (50 mL) and dichloromethane (50mL), successively. Compound **7** was afforded as a yellow solid (0.300 g, 52%). mp > 300 °C (lit. > 300 °C[1]). δ_H (500 MHz, DMSO) 12.96 (1 H, br, COO$\underline{H}$), 8.20 (1 H, d, *J* 8.5, $\underline{H}_{Ar}$), 8.03 – 7.98 (2 H, m, $\underline{H}_{Ar}$), 7.73 – 7.67 (1 H, m, $\underline{H}_{Ar}$), 4.58 (2 H, s, NC$\underline{H}_2$), 4.03 (3 H, s, NC$\underline{H}_3$). δ_C (125 MHz, DMSO) 169.3 ($\underline{C}$OOH), 159.1 (N$\underline{C}$O), 154.3 (N$\underline{C}$O), 149.7 ($\underline{C}_{Ar}$), 137.2 ($\underline{C}_{Ar}$), 135.5 ($\underline{C}_{Ar}$), 135.2 ($\underline{C}_{Ar}$), 133.5 ($\underline{C}_{Ar}$), 131.8 ($\underline{C}_{Ar}$), 126.4 ($\underline{C}_{Ar}$), 116.8 ($\underline{C}_{Ar}$), 42.4 (N$\underline{C}$H$_2$), 32.0 (N$\underline{C}$H$_2$). m/z (ESI) 309 ([M+Na]$^+$).

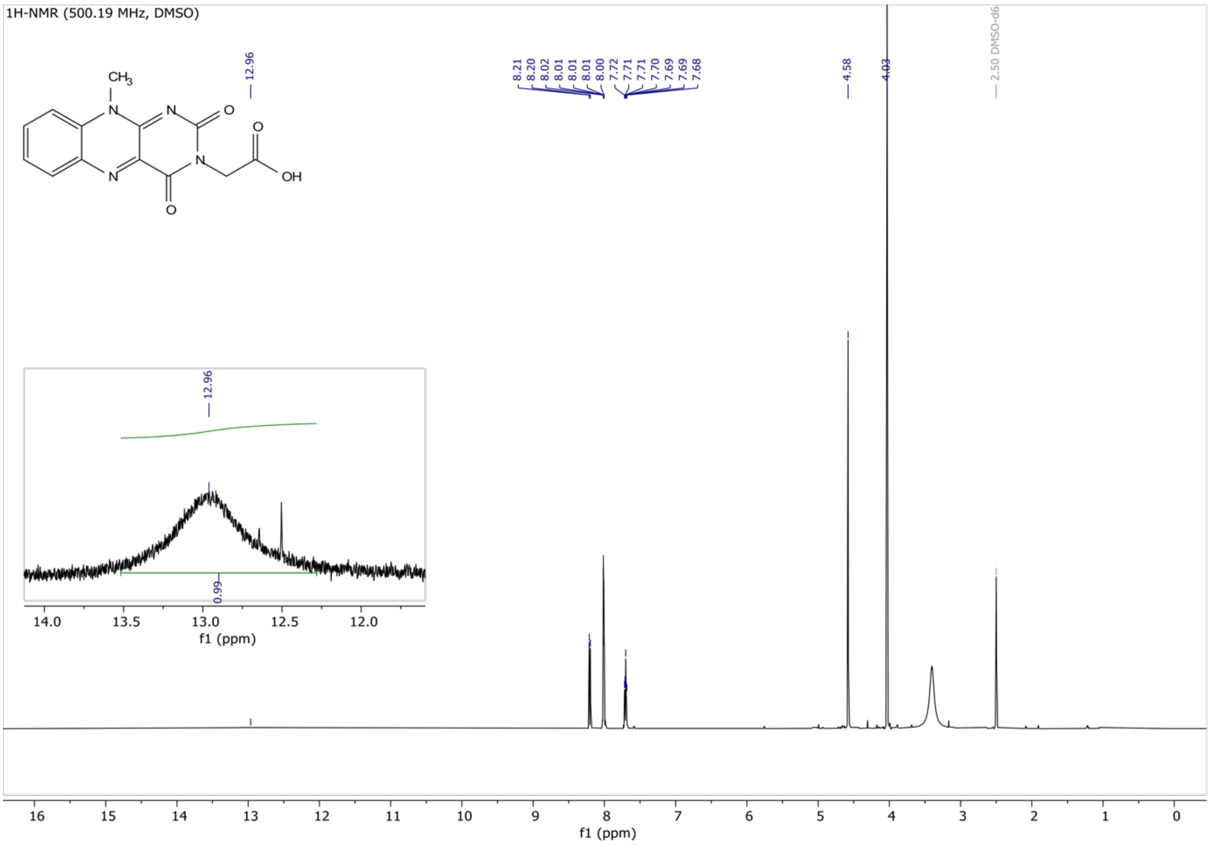

Figure S6. ^{1}H NMR spectrum of compound **7**.

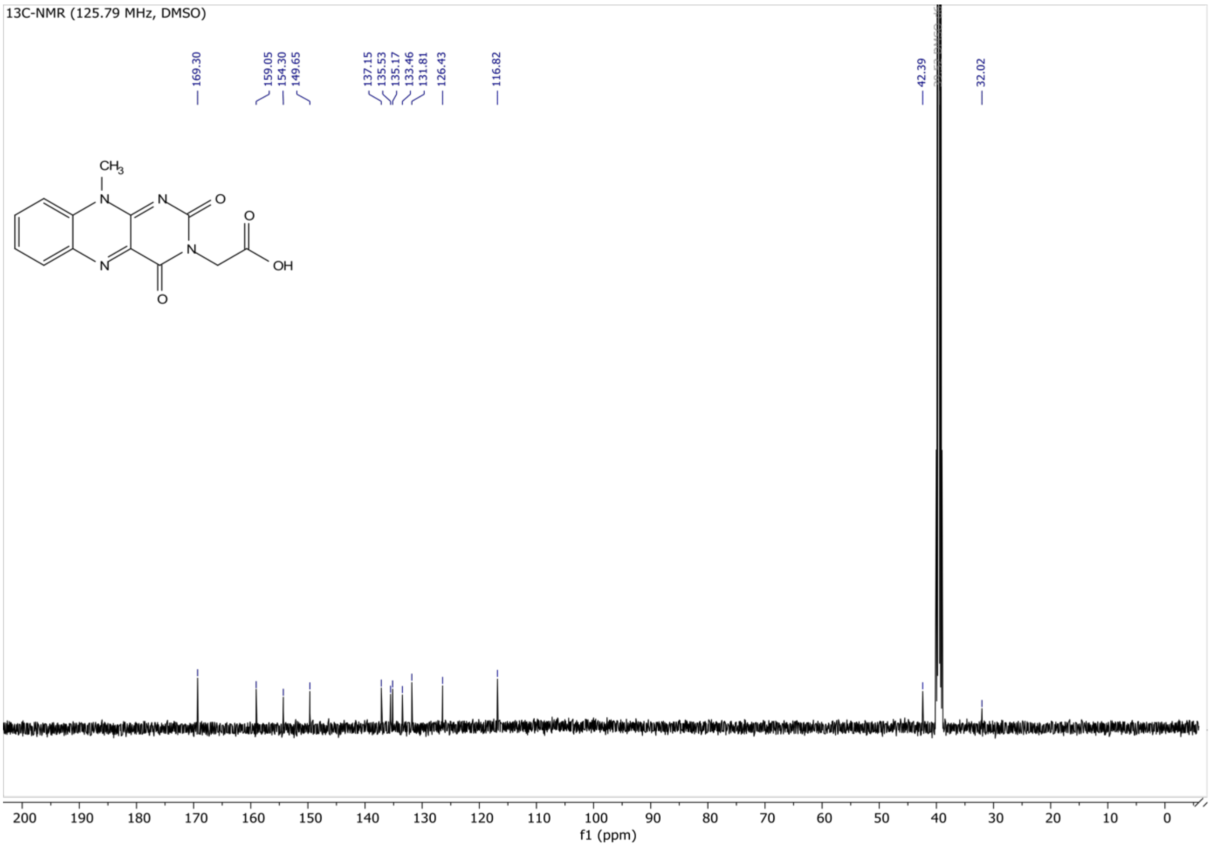

Figure S7. ^{13}C NMR spectrum of compound **7**.

tert-Butyl (2*S*)-2-[(2*S*)-2-{[(benzyloxy)carbonyl]amino}-3-phenylpropanamido]-3-phenylpropanoate (**1**)

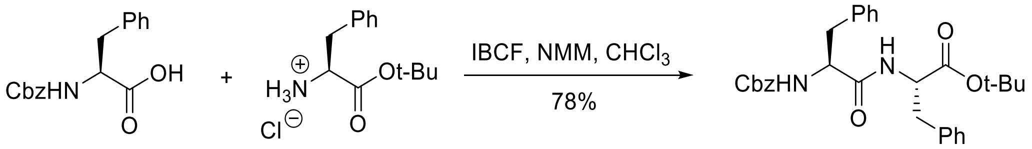

1

To a solution of *N*-Cbz-(*S*)-phenylalanine (1.41 g, 4.71 mmol) in chloroform (20 mL) was added isobutyl chloroformate (IBCF, 1 eq, 614 μL) and *N*-methylmorpholine (NMM, 1.1 equiv, 570 μL). The mixture was stirred for 10 minutes. (*S*)-Phenylalanine *tert*-butyl ester hydrochloride (1.02 eq, 1.24 g) was then added, followed by another portion of *N*-methylmorpholine (1.1 eq, 570 μL). After stirring overnight, the reaction mixture was diluted with chloroform, washed in turn with 1 M hydrochloric acid, water, and brine, dried ($MgSO_4$), and evaporated to dryness under reduced pressure. Crude **1** was thus obtained as a pale yellow oil (2.28 g) which solidified on standing. Column chromatography (1:99 ethyl acetate/dichloromethane, wet-loaded, *ca.* 5×4 cm) followed by removal of residual solvent by evaporation from acetonitrile afforded the title compound **1** as a colourless oil (1.85 g, 78%).

δ_H (400 MHz, DMSO-d_6) 8.42 (1H, d, *J* 7.36, N<u>H</u>), 7.50 (1H, d, *J* 8.89, N<u>H</u>), 7.35-7.06 (15H, m, <u>H</u>$_{Ar}$), 4.92 (2H, s, PhC<u>H</u>$_2$O), 4.41-4.33 (1H, m, C<u>H</u>*), 4.32-4.23 (1H, m, C<u>H</u>*), 3.01-2.89 (3H, m, PhC<u>H</u>$_2$CH), 2.69 (1H, dd, *J* 13.87, 11.37, PhC<u>H</u>$_2$CH), 1.31 (9H, s, C(C<u>H</u>$_3$)$_3$). δ_C (100 MHz, DMSO-d_6) 171.67, 170.41, and 155.81 (<u>C</u>=O), 138.09, 137.12, 137.03, 129.26, 129.22, 128.29, 128.19, 128.03, 127.68, 127.43, 126.53, and 126.26 (<u>C</u>$_{Ar}$), 80.67 (<u>C</u>(CH$_3$)$_3$), 65.17 (Ph<u>C</u>H$_2$O), 55.90 and 54.25 (<u>C</u>H*), 37.49 and 36.82 (Ph<u>C</u>H$_2$CH), 27.52 (C(<u>C</u>H$_3$)$_3$). HRMS (ESI) m/z: $[M+Na]^+$ calcd for $C_{30}H_{34}N_2NaO_5$ 525.2360; found 525.2346.

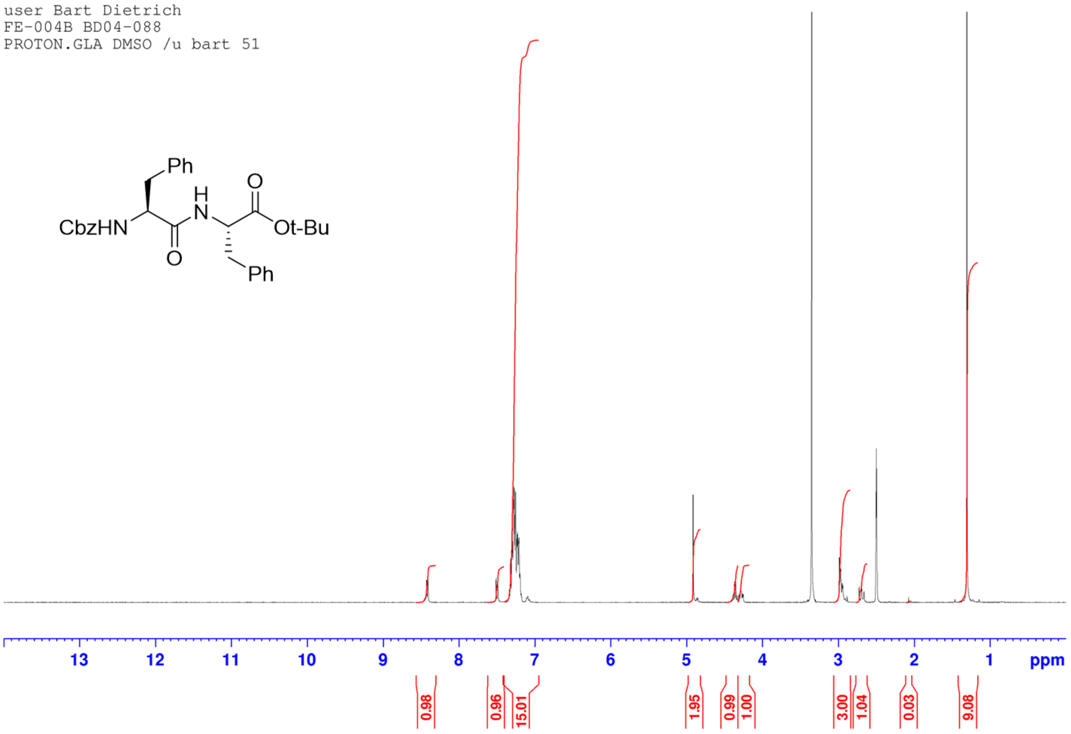

Figure S8. ^{1}H NMR spectrum of compound **1**.

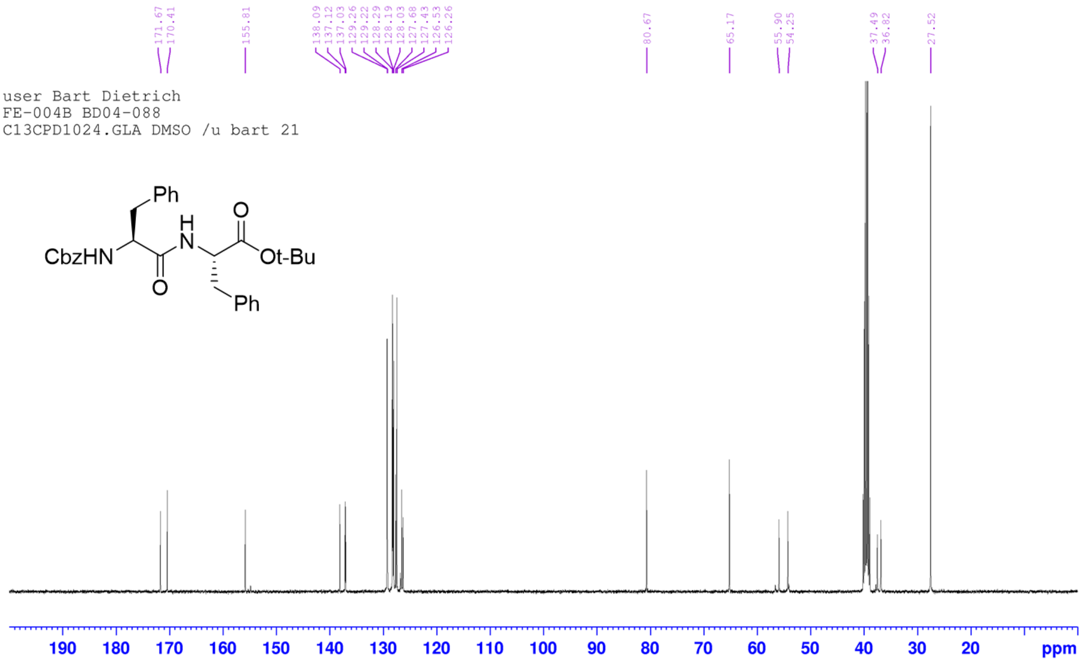

Figure S9. ^{13}C NMR spectrum of compound **1**.

tert-Butyl (2*S*)-2-[(2*S*)-2-amino-3-phenylpropanamido]-3-phenylpropanoate (**2**)

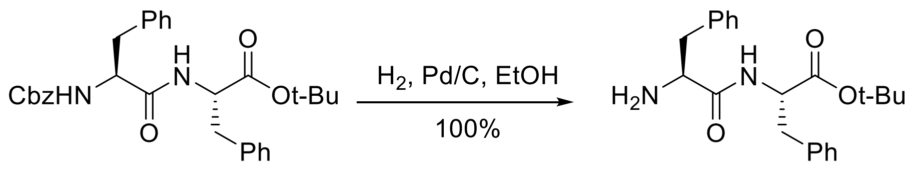

1 **2**

To a solution of **1** (1.81 g, 3.60 mmol) in ethanol (50 mL) was added 10% palladium on carbon (5 mol% in Pd, 192 mg) and the mixture was stirred under ≈1 bar H_2 pressure (balloon) overnight. After this time, TLC (1:9 ethyl acetate/dichloromethane) indicated the absence of starting material. The reaction mixture was filtered through celite and evaporated under reduced pressure. **2** was obtained as a viscous oil which solidified into a waxy solid on standing (1.32 g, 100%). NMR showed a small amount (< 1%) of residual ethanol.

δ_H (400 MHz, $CDCl_3$) 7.78 (1H, d, *J* 8.10, N<u>H</u>), 7.34-7.20 (8H m, $\underline{H}_{Ar}$), 7.11-7.08 (2H, m, $\underline{H}_{Ar}$), 4.78 (1H, ddd, *J* 8.30, 6.24, 6.24, C<u>H</u>*NH), 3.61 (1H, dd, *J* 9.37, 3.87, C<u>H</u>*NH_2), 3.18 (1H, dd, *J* 13.70, 3.81, PhC$\underline{H}_2$), 3.08 (2H, app. d, *J* 6.23, PhC$\underline{H}_2$), 2.60 (1H, dd, *J* 13.69, 9.38, PhC$\underline{H}_2$), 1.41 (11H, br s, C(C$\underline{H}_3$)$_3$ and N$\underline{H}_2$ overlapped by H_2O). δ_C (100 MHz, DMSO-d_6) 174.10 and 170.46 (<u>C</u>=O), 138.57, 136.91, 129.41, 129.29, 128.16, 128.10, 126.56, and 126.14 ($\underline{C}_{Ar}$), 80.88 (<u>C</u>(CH_3)$_3$), 55.80 (<u>C</u>H*), 53.46 (<u>C</u>H*), 40.74 (Ph<u>C</u>H_2), 37.25 (Ph<u>C</u>H_2), 27.54 (C(<u>C</u>H_3)$_3$). HRMS (ESI) m/z: [M+Na]$^+$ calcd for $C_{22}H_{28}N_2NaO_3$ 391.1992; found 391.1985.

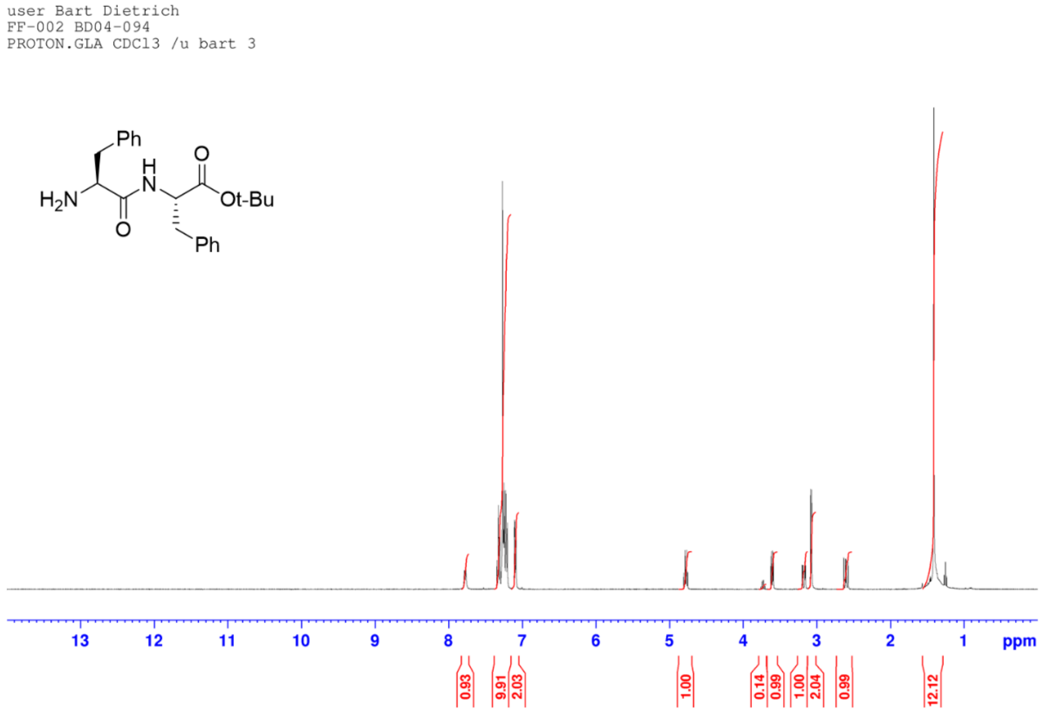

Figure S10. ^{1}H NMR spectrum of compound **2**.

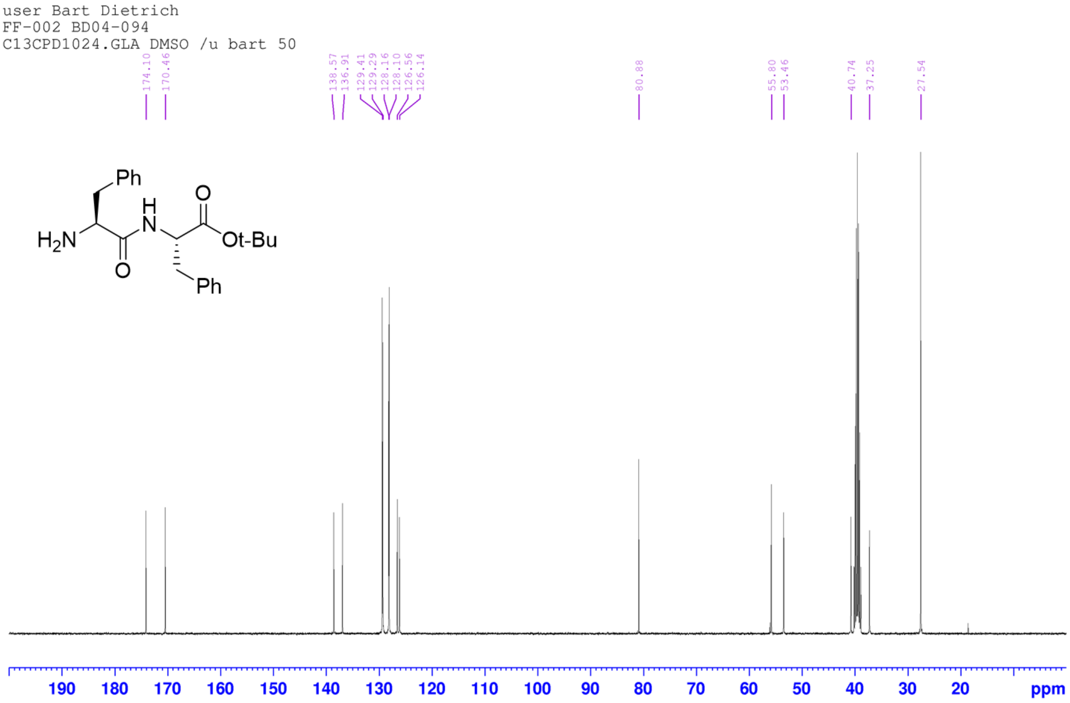

Figure S11. ^{13}C NMR spectrum of compound **2**.

<u>*tert*-Butyl (2*S*)-2-[(2*S*)-2-amino-3-phenylpropanamido]-3-phenylpropanoate hydrochloride (**3**)</u>

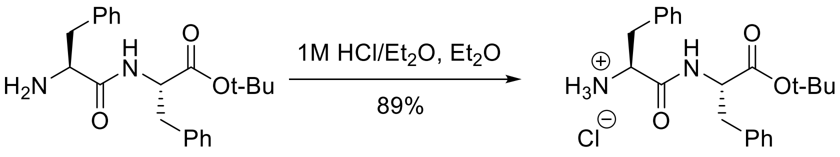

2 **3**

To a solution of **2** (1.25 g, 3.39 mmol) in diethyl ether (75 mL) was added hydrogen chloride in diethyl ether (1 eq, 3.39 mL of a 1M solution in diethyl ether diluted with a further 25 mL diethyl ether) dropwise with vigorous stirring. After complete addition the reaction mixture was stirred for a further 30 minutes. It was then filtered, the solid in the filter washed with diethyl ether, and then dried under vacuum. **3** was thus obtained as a white solid (1.22 g, 89%). NMR data shows a small amount (< 0.5%) of residual diethyl ether.

δ_H (400 MHz, DMSO-d$_6$) 9.19 (1H, d, *J* 7.37, N<u>H</u>), 8.21 (3H, br s, N<u>H</u>$_3^+$), 7.38-7.19 (10H, m, <u>H</u>$_{Ar}$), 4.41 (1H, pseudo-q, *J* 7.30, C<u>H</u>*), 4.09 (1H, dd, *J* 7.79, 5.19, C<u>H</u>*), 3.20 (1H, dd, *J* 14.04, 5.06, PhC<u>H</u>$_2$), 3.04-2.92 (3H, m, PhC<u>H</u>$_2$), 1.31 (9H, s, C(C<u>H</u>$_3$)$_3$). δ_C (100 MHz, DMSO-d$_6$) 169.95 and 168.10 (<u>C</u>=O), 136.88, 134.89, 129.74, 129.31, 128.35, 128.24, 127.04, and 126.62 (<u>C</u>$_{Ar}$), 80.97 (<u>C</u>(CH$_3$)$_3$), 54.59 and 53.11 (<u>C</u>H*), 36.96 and 36.73 (Ph<u>C</u>H$_2$), 27.48 (C(<u>C</u>H$_3$)$_3$). HRMS (ESI) m/z: [M-HCl+Na]$^+$ calcd for $C_{22}H_{28}N_2NaO_3$ 391.1992; found 391.1991.

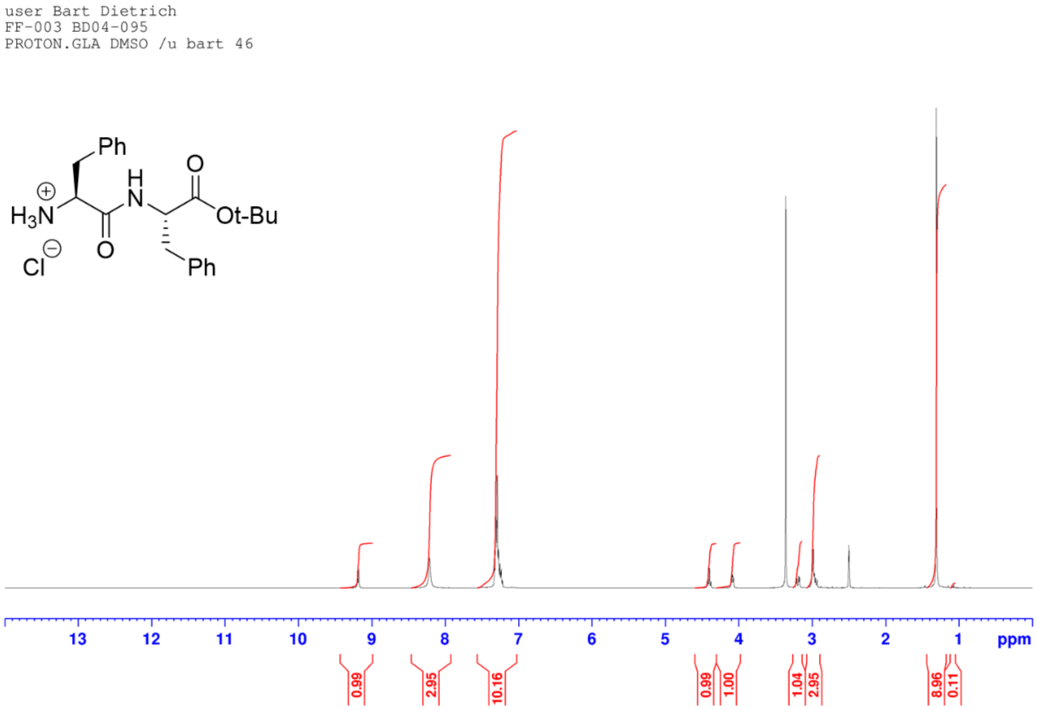

Figure S12. ^{1}H NMR spectrum of compound **3**.

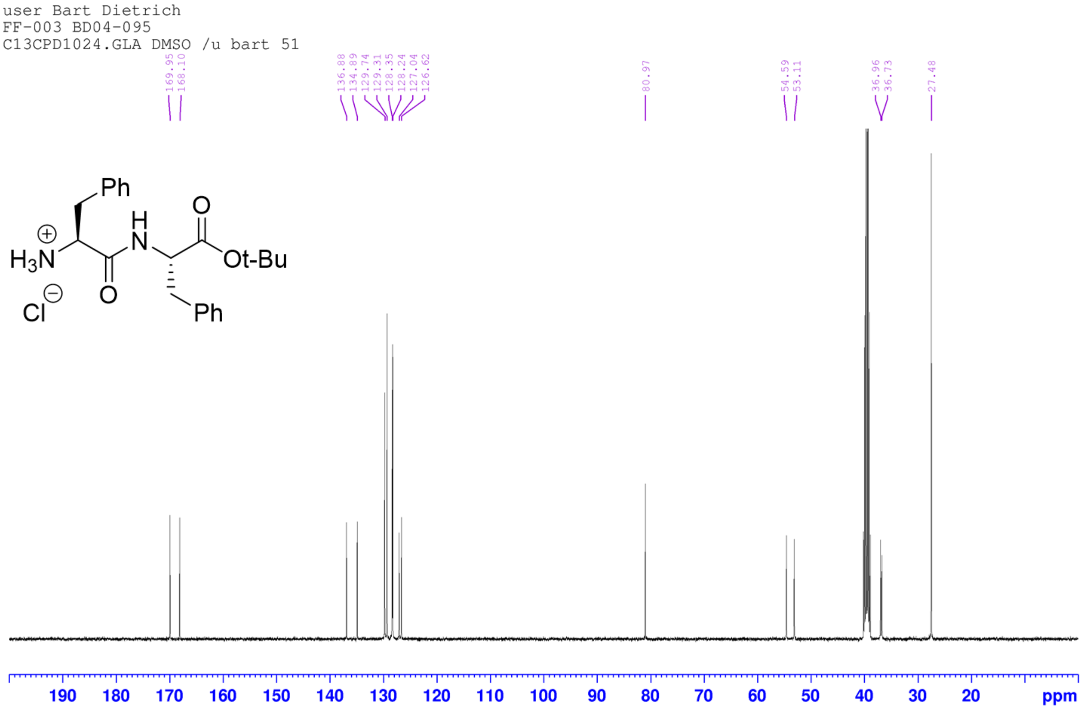

Figure S13. ^{13}C NMR spectrum of compound **3**.

tert-Butyl (2*S*)-2-[(2*S*)-2-(2-{10-methyl-2,4-dioxo-2*H*,3*H*,4*H*,10*H*-benzo[g]pteridin-3-yl}acetamido)-3-phenylpropanamido]-3-phenylpropanoate (**8**)

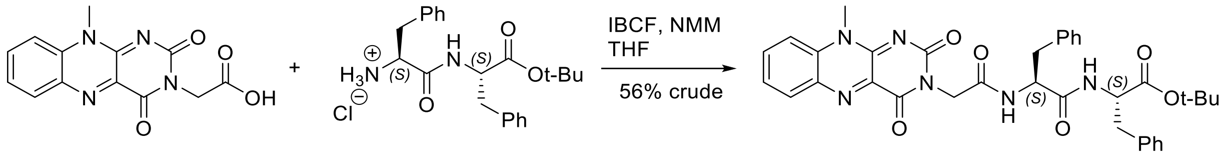

7 **3** **8**

Compound **7** (935 mg, 3.27 mmol) was suspended in tetrahydrofuran (45 mL) and *N*-methylmorpholine (1.1 eq, 396 μL) and isobutyl chloroformate (1.02 eq, 435 μL) were added. The mixture was stirred for 10 minutes, then **3** (1.01 eq, 1.34 g) and another portion of *N*-methylmorpholine (1.1 eq, 396 μL) were added and the reaction was stirred vigorously overnight. The reaction mixture was filtered, and the residue in the filter was washed with 1M hydrochloric acid, water, and then oven-dried at 60 °C, affording crude **8** as an ochre solid (1.16 g, 56%). **Note**: evaporation of the filtrate afforded a smaller amount (606 mg) of product of even lesser purity which was discarded. NMR analysis suggest contamination with unreacted starting materials and attempts at purification by column chromatography or recrystallisation were met with limited success. The crude compound was used directly in the following step. ^{1}H NMR data below indicate expected integrations and ignore contributions of contaminants. No carbon NMR or mass spectral data were collected.

δ_H (400 MHz, DMSO-d_6) 8.44 (1H, d, *J* 7.28, N<u>H</u>), 8.34 (1H, d, *J* 8.40, N<u>H</u>), 8.23-8.17 (1H, m, $\underline{H}_{Ar}$), 8.03-7.98 (2H, m, $\underline{H}_{Ar}$), 7.73-7.65 (1H, m, $\underline{H}_{Ar}$), 7.32-7.16 (10H, m, $\underline{H}_{Ar}$), 4.62-4.55 (1H, m, C<u>H</u>*), 4.48 (2H, s, $NC\underline{H}_2$), 4.35 (1H, dd, *J* 14.77, 7.44, C<u>H</u>*), 4.03 (3H, s, $NC\underline{H}_3$), 3.04-2.93 (3H, m, $PhC\underline{H}_2$), 2.76 (1H, dd, *J* 13.76, 9.11, $PhC\underline{H}_2$), 1.92 (9H, s, $C(C\underline{H}_3)_3$).

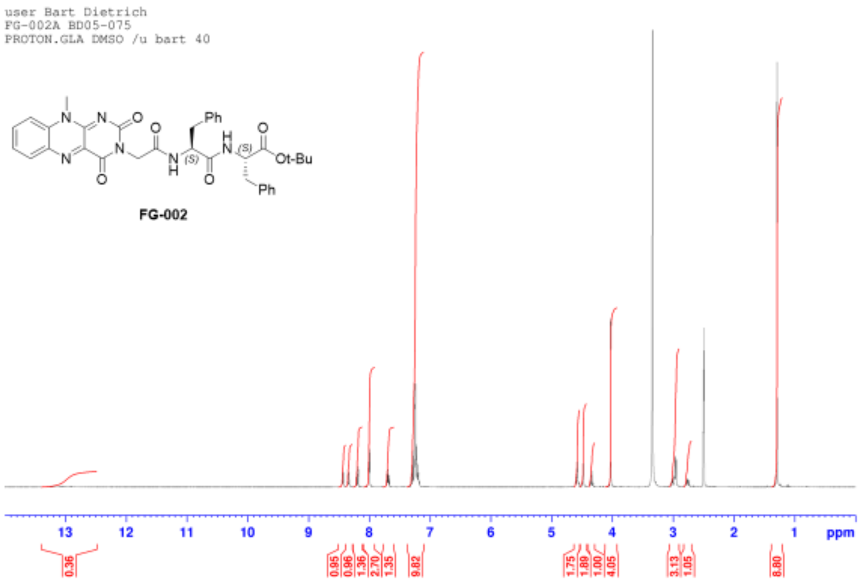

Figure S14. crude ^{1}H NMR spectrum of compound **8**.

(2*S*)-2-[(2*S*)-2-(2-{10-Methyl-2,4-dioxo-2*H*,3*H*,4*H*,10*H*-benzo[g]pteridin-3-yl}acetamido)-3-phenylpropanamido]-3-phenylpropanoic acid (**Fla-FF**)

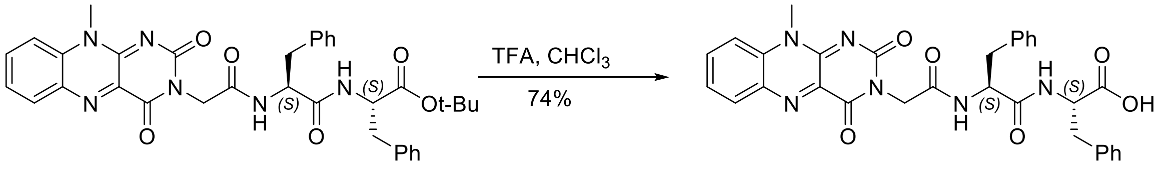

8 **Fla-FF**

To a suspension of crude **8** (1.16 g, 1.82 mmol) in chloroform (20 mL) was added trifluoroacetic acid (*ca.* 70 eq, 10 mL) and the mixture was stirred overnight. After this time, the reaction mixture was concentrated under reduced pressure to remove most of the chloroform. To the viscous residue was added diethyl ether (*ca.* 100 mL) and the mixture was stirred for 2 hours then filtered. The residue was washed with diethyl ether and recrystallised from boiling ethanol. **Fla-FF** was thus obtained as an orange-brown solid (785 mg, 74%).

δ_H (400 MHz, DMSO-d_6) 12.72 (1H, br s, COO$\underline{H}$), 8.31 (1H, d, *J* 6.92, N$\underline{H}$), 8.30 (1H, d, *J* 7.91, N$\underline{H}$), 8.21-8.19 (1H, m, $\underline{H}_{Ar}$), 8.02-7.97 (2H, m, $\underline{H}_{Ar}$), 7.73-7.66 (1H, m, $\underline{H}_{Ar}$), 7.32-7.14 (10H, m, $\underline{H}_{Ar}$), 4.56 (1H, ddd, *J* 8.53, 8.52, 4.50, C$\underline{H}$*), 4.51 (1H, d, *J* 16.30, NC$\underline{H}_2$), 4.46 (1H, d, *J* 16.34, NC$\underline{H}_2$), 4.43 (1H, ddd, *J* 8.04, 8.04, 5.36, C$\underline{H}$*), 4.03 (3H, s, NC$\underline{H}_3$), 3.06 (1H, dd, *J* 13.93, 5.46, PhC$\underline{H}_2$), 2.99 (1H, dd, *J* 13.90, 4.62, PhC$\underline{H}_2$), 2.93 (1H, dd, *J* 13.97, 8.63, PhC$\underline{H}_2$), 2.75 (1H, dd, *J* 13.83, 8.86, PhC$\underline{H}_2$). δ_C (100 MHz, DMSO-d_6) 172.67, 170.89, 166.51, 159.07, and 154.44 ($\underline{C}$=O), 149.43, 137.62, 137.40, 137.35, 135.40, 134.92, 133.22, 131.69, 129.30, 129.12, 128.27, 128.03, 126.48, 126.36, 126.22, and 116.67 ($\underline{C}_{Ar}$), 53.64 (both $\underline{C}$H* overlapping), 43.46 (N$\underline{C}H_2$), 37.52 and 36.75 (Ph$\underline{C}H_2$), 31.90 (N$\underline{C}H_3$). HRMS (ESI) m/z: $[M+Na]^+$ calcd for $C_{31}H_{28}N_6NaO_6$ 603.1963; found 603.1973, $[M+2Na-H]^+$ calcd for $C_{31}H_{27}N_6Na_2O_6$ 625.1793; found 625.1806.

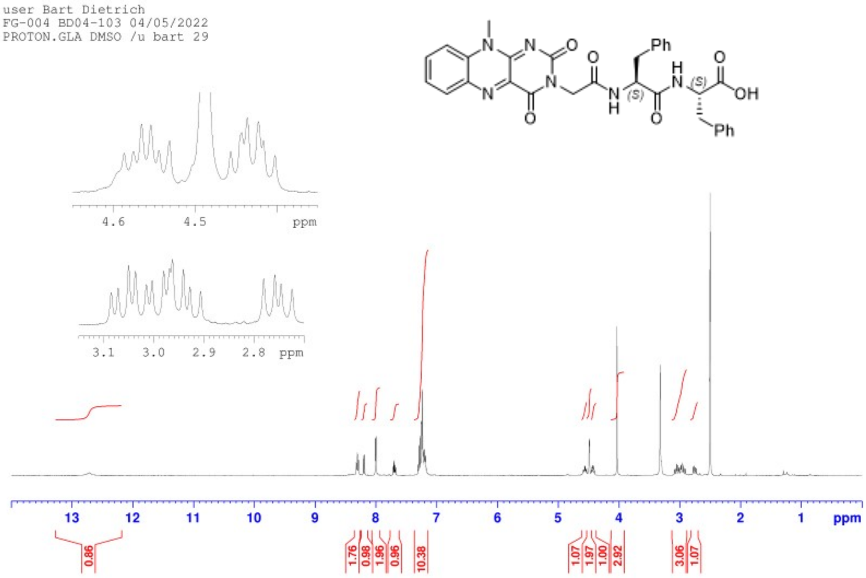

Figure S15. ^{1}H NMR spectrum of **Fla-FF**. Insets show the amino acid α-proton (top) and benzylic proton (bottom) regions. The lack of asymmetry in the α-proton signals (note that the NC$\underline{H}_2$ signal is two highly roofed doublets, the distal peaks of which distort the symmetry of the α-proton signals somewhat), and the tidy appearance of the benzylic region (exactly 16 peaks as expected from four doublet-of-doublets) suggest lack of racemisation.[3]

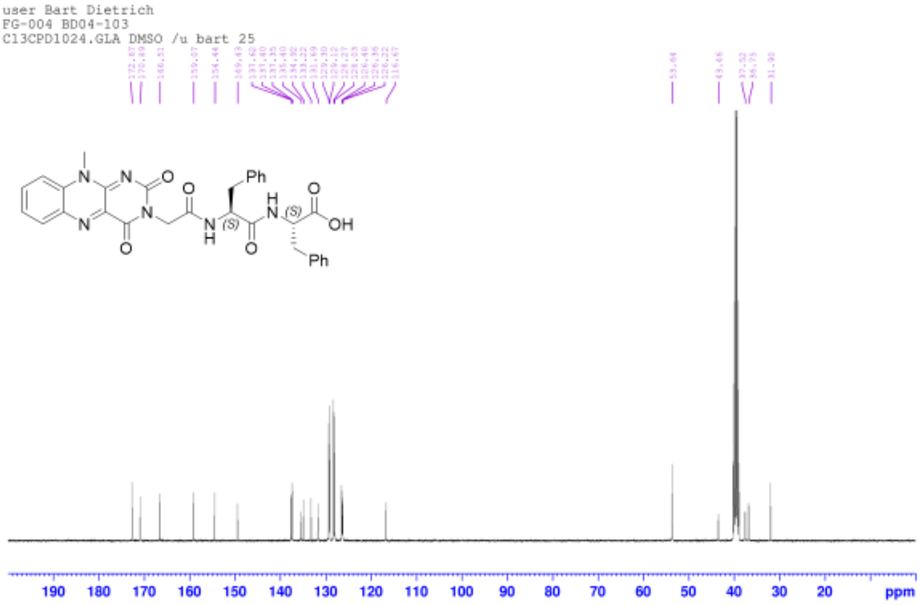

Figure S16. ^{13}C NMR spectrum of **Fla-FF**.

Small Angle X-ray Scattering (SAXS). SAXS data were collected on a Ganesha 300XL instrument (Xenocs). Samples were loaded into 3.5mm borosilicate glass capillaries (Capillary Tube Supplies Ltd). The capillaries were sealed using fast-curing two-part epoxy (Araldite) and left to set for at least 15 minutes. SAXS data were collected at room temperature over a Q range of 0.007 – 0.25Å^{-1} for an exposure time of 3600 seconds. All measurements were corrected for transmission and absolute intensity and had the solvent background and empty capillary scattering subtracted before processing. Data were reduced using SAXSGUI, and model fits were performed using SASView 4.0.[4]

Table S1. Summary of fits to SAXS data.

**Sample**	**Solution**	**pH triggered gel**	**Solvent-triggered gel**	**Low pH sol**
Model	Flexible cylinder	Flexible elliptical cylinder	Flexible elliptical cylinder	Flexible cylinder and power law
Scale	0.00018 ± 2.1 x 10^{-5}	0.00025 ± 1.2 x 10^{-5}	2.88e-05 ± 2.1 x 10^{-6}	2.97 ± 0.4
Background	0.0091 ± 0.0004	0.026 ± 0.0005	0.013 ± 0.0005	0.017 ± 0.0009
Length	>1000	>1000	>1000	>1000
Kuhn Length	58.4 ± 19.5	151.4 ± 0.05	337.2 ± 12.5	391.7 ±
Radius	30.5 ± 1.8	25.0 ± 0.8	57.0 ± 1.1	91.6 ±
Axis Ratio		3.0 ± 0.1	9.07 ± 0.7	
Power Law Scale				1.2 ± 0.2
Power Law				2.59 ± 0.1
Chi^2	1.34	1.39	2.60	5.88

Table S1. Parameters from fits to SAXS data shown in main text Figure 2, and also discussed during the photooxidation. In all cases, the number on top is the fit value and the number below the error provided by the fitting software.

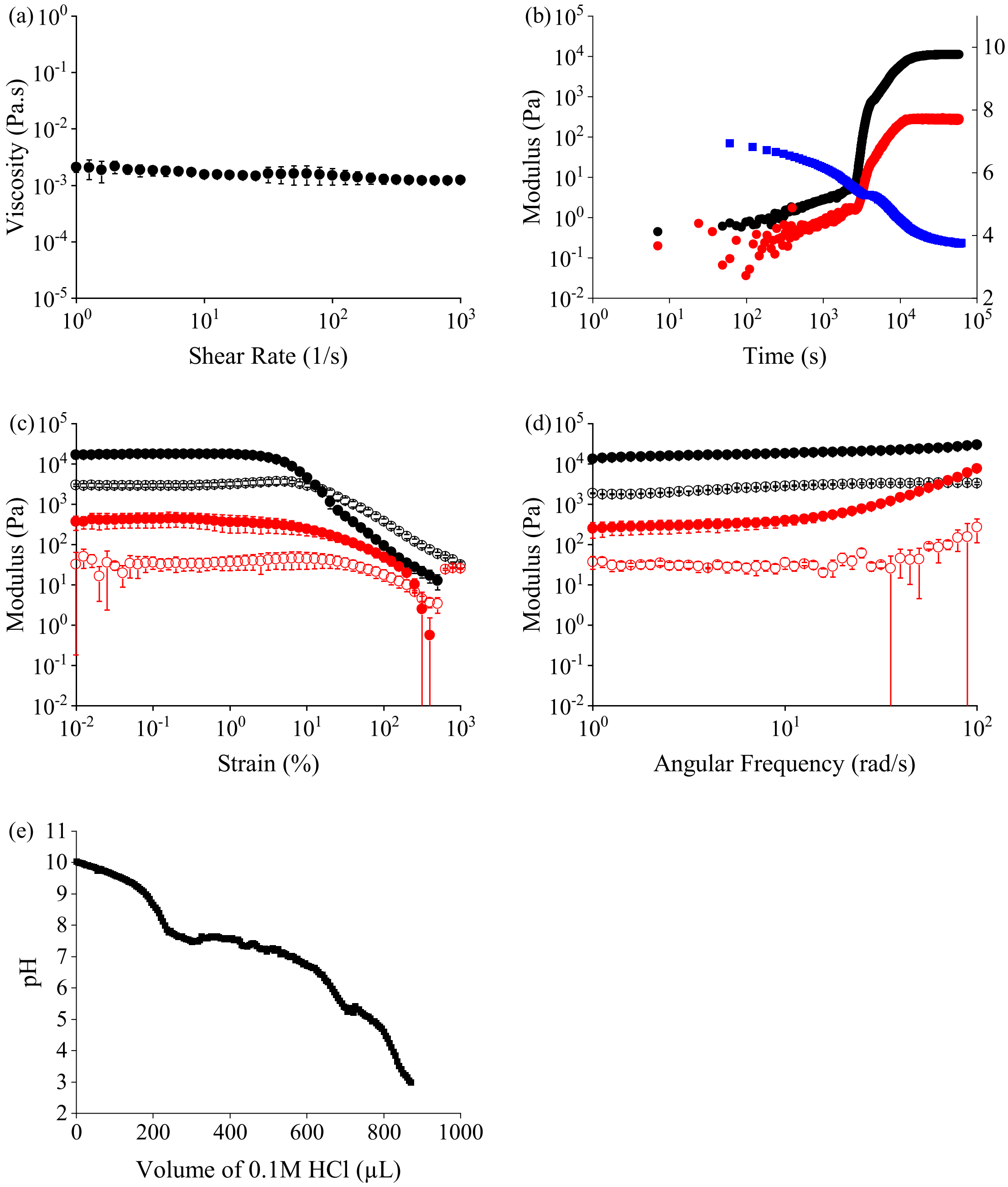

Figure S17. All pH and rheology data for 5 mg/mL **Fla-FF** triggered by pH or solvent. (a) Viscosity of 5 mg/mL **Fla-FF** at pH 9. Measurements were performed in triplicate with error bars showing the standard deviation between the samples. (b) Overnight time sweep and pH showing G′ (black) and G″ (red) growing with time whilst pH (right axis, blue) decreases. Strain (c) and frequency (d) data for 5 mg/mL **Fla-FF** gels, triggered by pH (black) and solvent (red). G′ is represented by filled circles and G″ by empty circles. (e) Apparent pK_a titration for 5 mg/mL **Fla-FF**.

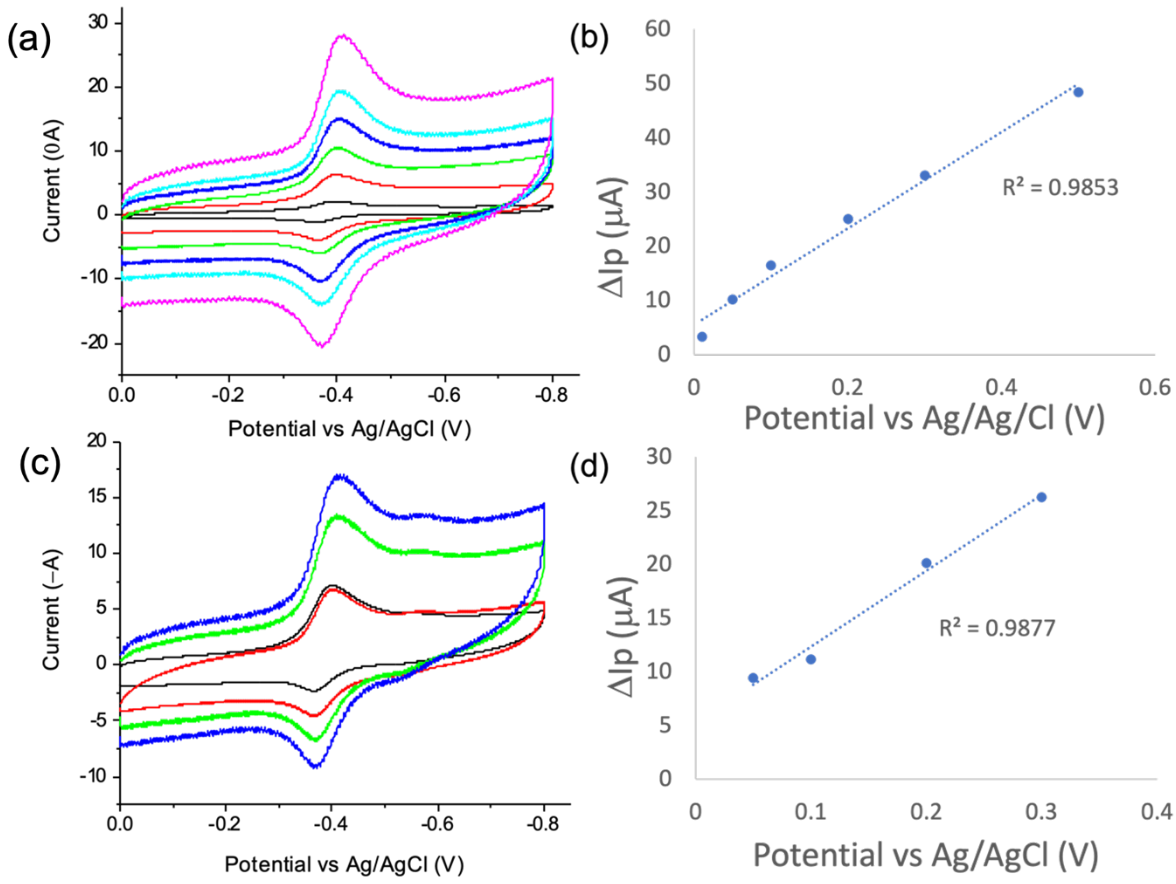

Figure S18. (a) CV traces of **5** at pH 7, at different potential sweep rates; (b) Plot of the peak current difference as a function of the potential sweep rate for **5**; (c) CV traces of **Fla-FF** at pH 7, at different potential sweep rates; (d) Plot of the peak current difference for **Fla-FF** as a function of the potential sweep rate.

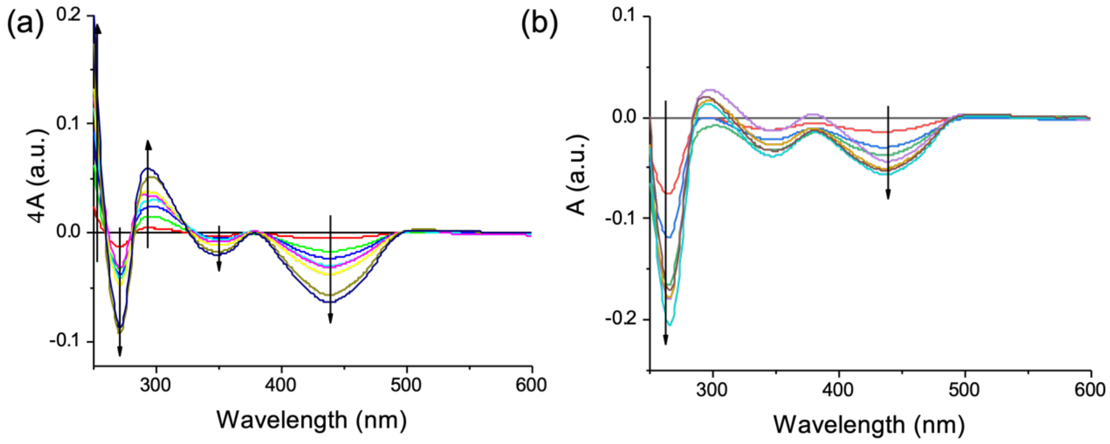

Figure S19. (a) SPEC traces of **Fla-FF** in water at pH 9, displayed as difference plots with the trace of the absorption at no applied potential. A reduction potential of ΔV = 0.1 V is progressively applied; (b) SPEC traces of **Fla-FF** in water at pH 3, displayed as difference plots with the trace of the absorption at no applied potential. A reduction potential of ΔV = 0.1 V is progressively applied.

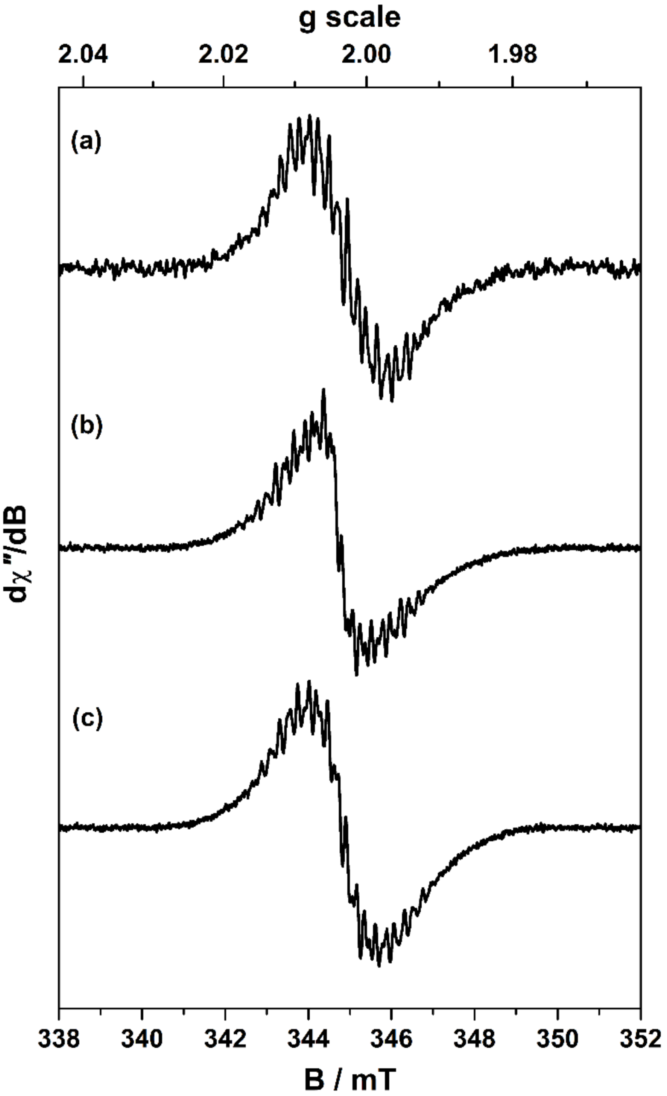

Figure S20. X-band EPR spectra of flavin radical formed by **Fla-FF** in (a) solution, (b) pH-triggered gel and (c) DMF gel generated by irradiation at 450 nm at ambient temperature (experimental conditions: frequency, 9.667 GHz; power, 1 mW; modulation, 0.1 mT).

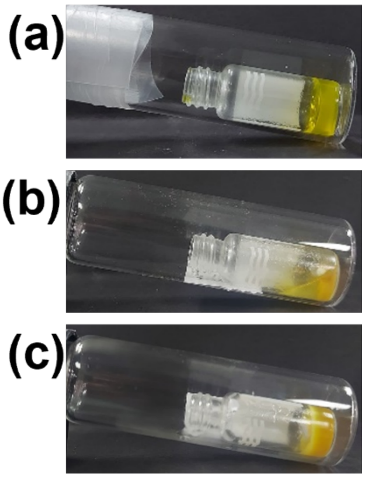

Figure S21. (a) **Fla-FF** gel prepared with HCl before photocatalysis. (b) **Fla-FF** gel prepared with HCl after photocatalysis with thioanisole. (c) **Fla-FF** gel prepared with HCl after photocatalysis with cyclo-hexyl methyl sulfide.

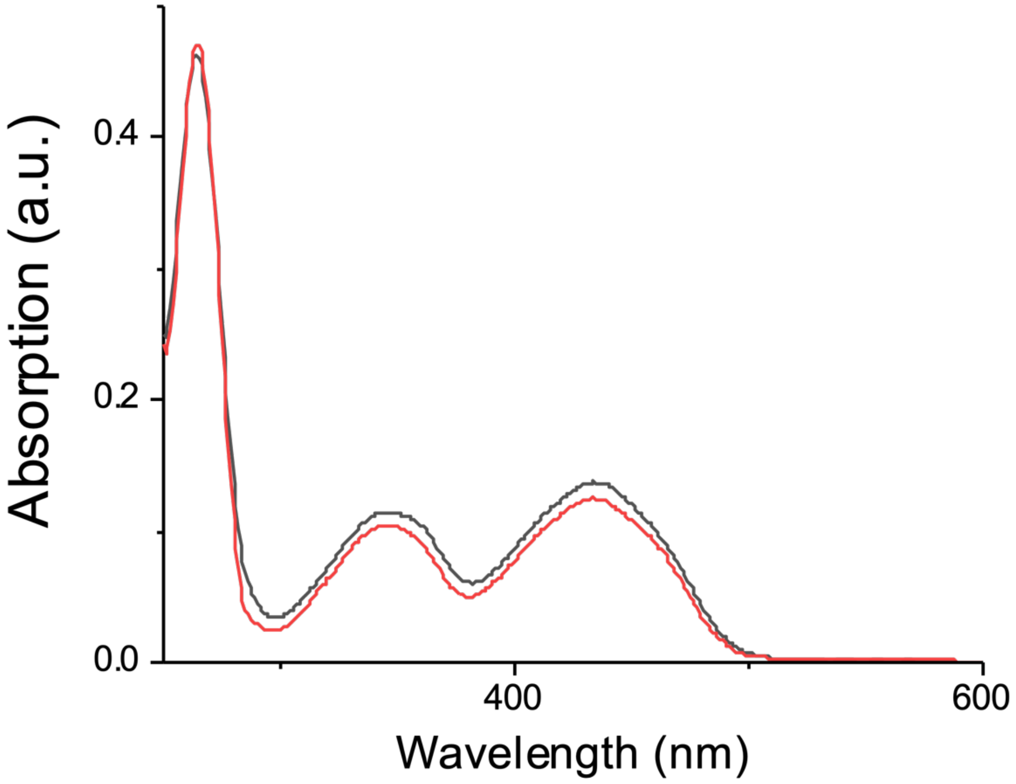

Figure S22. UV-Vis spectra of **Fla-FF** (black) and **7** (red) at a concentration of 1.75 x 10^{-2} M at pH 9 in water. 0.01 mm path length cuvettes were used to collect the data.

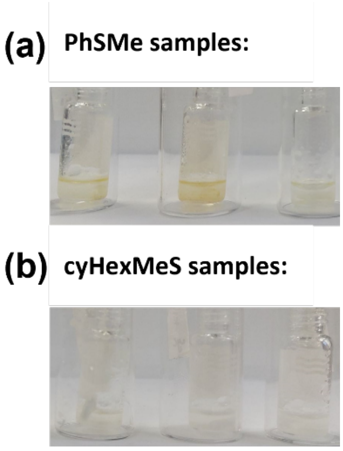

Figure S23. Samples of Flavin **7** pH 9 sol with (a) PhSMe and (b) cyHexMeS after photocatalysis reaction.

Table S2. Control experiments for photooxidation reactions using Fla-FF photocatalyst.

[a] - no light. [b] – Argon atmosphere. [c] – no Fla-FF. BQ = benzoquinone. DPBF = 1,3diphenylisobenzofuran. * = internal standard NMR yield.

	Substrate	Additive	Sulfoxide	Sulfone	1,2-dibenzoylbenzene
**Fla-FF** (pH 9)	PhSMe[a]	-	0%	0%	-
**Fla-FF** (pH 9)	PhSMe[b]	-	2%	0%	-
**-**	PhSMe[c]	-	0.5%	0%	-
**Fla-FF** (pH 9)	PhSMe	BQ	31%	0%	-
**Fla-FF** (pH 4, sol B)	PhSMe	BQ	40%	0%	-
**Fla-FF** (pH 4, gel A)	PhSMe	BQ	29%	0%	-
**Fla-FF** (pH 9)	cyHexSMe	BQ	31%	0%	-
**Fla-FF** (pH 4, sol B)	cyHexSMe	BQ	40%	0%	-
**Fla-FF** (pH 4, gel A)	cyHexSMe	BQ	34%	0%	-
**Fla-FF** (pH 9)	DPBF	-	-	-	26%*
**Fla-FF** (pH 4, sol B)	DPBF	-	-	-	41%*
**Fla-FF** (pH 4, gel A)	DPBF	-	-	-	48%*
**Fla-FF** (pH 9)	PhSMe	NaN_3	64%	5%	-
**Fla-FF** (pH 9)	cyHexSMe	NaN_3	43%	18%	-

References

1. M. Á. Farrán, R. M. Claramunt, C. López, E. Pinilla, M. R. Torres and J. Elguero, *ARKIVOC*, 2006, **2007**, 20-38.
2. J. Takeda, S. Ota and M. Hirobe, *Journal of the American Chemical Society*, 1987, **109**, 7677-7688.
3. K. McAulay, B. Dietrich, H. Su, M. T. Scott, S. Rogers, Y. K. Al-Hilaly, H. Cui, L. C. Serpell, Annela M. Seddon, E. R. Draper and D. J. Adams, *Chemical Science*, 2019, **10**, 7801-7806.
4. www.sasview.org/.
